# Using semantics to scale up evidence-based chemical risk-assessments

**DOI:** 10.1371/journal.pone.0260712

**Published:** 2021-12-15

**Authors:** Catherine Blake, Jodi A. Flaws

**Affiliations:** 1 School of Information Sciences, University of Illinois at Urbana-Champaign, Urbana, Illinois, United States of America; 2 Department of Comparative Biosciences, University of Illinois at Urbana-Champaign, Urbana, Illinois, United States of America; Virginia Commonwealth University, UNITED STATES

## Abstract

**Background:**

The manual processes used for risk assessments are not scaling to the amount of data available. Although automated approaches appear promising, they must be transparent in a public policy setting.

**Objective:**

Our goal is to create an automated approach that moves beyond retrieval to the extraction step of the information synthesis process, where evidence is characterized as supporting, refuting, or neutral with respect to a given outcome.

**Methods:**

We combine knowledge resources and natural language processing to resolve coordinated ellipses and thus avoid surface level differences between concepts in an ontology and outcomes in an abstract. As with a systematic review, the search criterion, and inclusion and exclusion criterion are explicit.

**Results:**

The system scales to 482K abstracts on 27 chemicals. Results for three endpoints that are critical for cancer risk assessments show that refuting evidence (where the outcome decreased) was higher for cell proliferation (45.9%), and general cell changes (37.7%) than for cell death (25.0%). Moreover, cell death was the only end point where supporting claims were the majority (61.3%). If the number of abstracts that measure an outcome was used as a proxy for association there would be a stronger association with cell proliferation than cell death (20/27 chemicals). However, if the amount of supporting evidence was used (where the outcome increased) the conclusion would change for 21/27 chemicals (20 from proliferation to death and 1 from death to proliferation).

**Conclusions:**

We provide decision makers with a visual representation of supporting, neutral, and refuting evidence whilst maintaining the reproducibility and transparency needed for public policy. Our findings show that results from the retrieval step where the number of abstracts that measure an outcome are reported can be misleading if not accompanied with results from the extraction step where the directionality of the outcome is established.

## Introduction

The current methods used to conduct chemical risk assessments do not scale to recent regulatory changes such as the European Union’s REACH initiative that dramatically increases the number of chemicals to be assessed and the US EPAs trend towards cumulative risk assessments that consider multiple chemicals or combinations of chemical and non-chemical stressors. Manual processes used to synthesize evidence include 4 steps—retrieval, extraction, verification, and analysis [1]. Systematic reviews must include the search criterion, the databases searched and the search terms used [2, 3], and the inclusion and exclusion criterion to make the review scope clear to a reader, enable others to replicate or extend the work, and to instill trust by making it difficult to cherry-pick results. Automated systems for risk assessment have been developed to accelerate the retrieval step of the information synthesis process [4, 5]; however systems that employ black-box machine learning are not ideal in a public policy setting because it can be unclear why an abstract has been retrieved and because most system reports do not provide explicit inclusion and exclusion criteria.

Automated systems that identify relevant studies should not be confused with systems that extract, verify, and analyze the results from those studies. The latter steps in the review process differentiate studies that find an effect, from studies that find no-effect, and studies that refute the hypothesis that there is an effect. For example, an abstract that states “Furthermore, evidence is presented that AHTN is not genotoxic, does not induce peroxisome proliferation” [6] was labeled as indirect genotoxic peroxisome proliferation [4, 5], but this study refutes the hypothesis that the chemical was genotoxic and induces peroxisome proliferation. The authors of the previous work are clear that the system “does not exclude abstracts with no-effect results” [7], but this lack of differentiation between studies that find an association from those that do not is a major gap between automated approaches and manual efforts that attempt to include all evidence and then quantify the amount of supporting and refuting evidence. Thus stating that “a significant difference can be seen with higher numbers of abstracts for melanoma, reflecting the existing knowledge about the metastatic potential of melanoma” [8] conflates the number of abstracts that measure an outcome with those that find an association with melanoma because abstracts that do not find an association are not removed from the total number of abstracts retrieved. Similarly, consider the stated objective of a text mining approach to create a blood exposome database: “We aimed to generate a comprehensive blood exposome database of endogenous and exogenous chemicals associated with the mammalian circulating system through text mining and database fusion.” [9]. This could be easily misinterpreted as the number of chemicals that are positively associated, but the system does not analyze the direction of the association, but rather just retrieves studies that mention the mammalian circulating system.

Our goal is to bridge the gap between manual and automated approaches to systematically review literature on chemicals for risk assessments so that assessments can consider a greater number of chemicals and exposure sources. Such tools would reduce the time needed to conduct or reassess an individual or cumulative risk assessment as new information becomes available. Further, such tools can be used by scientific researchers to help focus their research questions based on existing literature.

In keeping with the definition of a systematic review the search criterion is explicit and definitions of each target outcome are provided. The proposed knowledge-based approach is consistent with manual processes that include explicit inclusion and exclusion criterion. Synonyms from the Unified Medical Language System (UMLS) are collected and abstracts are preprocessed using natural language processing (NLP) to manage coordinated ellipses [10] to avoid mismatches between the terms used and the text descriptions. For example, the NLP preprocessing identifies cell proliferation from sentence 1 and necrotic cell death from sentence 2 that would have otherwise been missed as the words in each of these phrases do not appear consecutively in the text. Once each target outcome is identified, explicit and observational claims from the Claim Framework [11] are used to characterize the evidence as supporting, where there is an increase (e.g. sentence 1), neutral where a change is reported but the directionality of the change is not provided (e.g. sentence 2). Refuting evidence, where there a decrease is reported, and negation are also identified for all claims, such as in sentence 3 that contains negated refuting evidence.

1. Example sentence with supporting evidence: A significant increase in regulated cell death and proliferation in salmon fed …”. (PMID 11255104).
2. Example sentence with neutral evidence: Cd(2+) caused necrotic and apoptotic cell death. (PMID 15665557)
3. Example sentence with negated refuting evidence: Neither sulindac sulfide nor sulfone inhibited cell proliferation under conditions where the drugs were growth inhibitory. (PMID 7606732)

Lastly, the spectrum of evidence from refuting to neutral to supporting is shown in a waffle plot that provides decision makers with both the amount of literature available and the directionality of that evidence. We demonstrate the benefit of moving from the retrieval to the extraction and analysis stages of the systematic review process using a set of 27 xenobiotic chemicals that are relevant to human exposure paradigms, known carcinogens, known endocrine disrupting chemicals, and/or known toxicants [12–15]. These chemicals are typical of those used by toxicologists and other scientists when conducting research on chemical exposures and cancer outcomes to guide research questions and experimental designs as well as to develop risk/benefit assessments. The target outcomes of cell death and cell proliferation together indicate one of the hallmarks of cancer [16] and because many of the selected chemicals are known endocrine disruptors and/or toxicants and studies indicate that endocrine disrupting chemicals often exert their toxic effects by interfering with proliferation and/or cell death.

## Related work

The target entities in this paper–cell death, cell proliferation and cells–appear in 21, 33 and 97 biomedical vocabularies respectively (https://bioportal.bioontology.org/). For example, the Gene Ontology (GO) [17] captures cell death and cell proliferation as biological processes and cells as a cellular components. The entities also appear in labeled text collections that were created to drive the development of automated information extraction tools such as the shared tasks on Gene Regulation Ontology (GRO) [18] and the Cancer Genetics task [19] that included cell death and cell proliferation. The GENIA collection comprises 2,000 abstracts on transcription factors for human blood cells and includes manual annotations for cell, cell types, cell components, and cell lines [20] and the CRAFT corpus comprises 97 full text articles on mouse genes [21] and includes manual annotations for multiple ontologies that capture our target entities. In contrast to the biomedical search terms for existing text corpora we provide the exact search string used to select the abstracts for each of the 27 chemicals that form our collection of 482,314 abstracts.

Many systems detect biomedical entities automatically (see [22] for a review). Such systems employ a knowledge-based approach such as MetaMap [23] where the system searches for expressions from an existing vocabulary (e.g. the exact phrase ‘cell death’), or a machine learning approach where a model is induced from training data. Automated approaches can be further characterized into those that employ traditional machine learning algorithms such as Naïve Bayes classifiers, or Support Vector Machines [24] and those that use a neural networks such as deep learning. In this work we extend the knowledge-based approach by using natural language processing to overcome surface level differences between the concept representations used by authors and how concepts are captured in a knowledge resource. Deep learning is also used to classify result or conclusion sentences to avoid including an author’s motivation or stated hypothesis with the outcomes of a study.

This work also relates to argumentation in biomedicine such as the Claim Framework [11] that captures how scientists who conduct empirical research report their findings. The Framework was developed by analyzing full text articles and comprises five types of claims: explicit, implicit, comparison, associations and correlations. Explicit claims are the most prevalent and require that a sentence include two entities (an entity that has been changed and an entity that is responsible for the change), and how the first entity changes the second such as in the sentence ‘The [CaN inhibitor cyclosporine A (CsA)]_entity1_ reduced_[change]_ [cell growth]_entity2._’. Observations are also included in this analysis where authors report a changed entity but do not include the entity that was responsible for the change.

Explicit claims are equivalent to the causal claim in [25]. The post error analysis found that the text needed to address a query appeared at the end of the abstract, which suggests that sentences were likely from the result and conclusion sections. In contrast to argumentation systems that strive to identify major claims [25] or to differentiate between major and minor claims, which has been shown to have low inter-rater reliability [26] we make no judgments regarding the veracity of a claim. Instead, directionality and negation of each claim are show to the decision maker as six discrete steps from refuting to negated refute, neutral, negated neutral, negated support, and finally to supporting evidence.

Other work that has contrasted supporting and refuting evidence has framed the task as identifying contradictions [25, 27]; however, neutral changes such as ‘Results showed a change in cell death’ do not fit into this framework. Moreover, ‘There was no significant increase in cell proliferation’ could mean that there was no change or that there was an increase that did not reach statistical significance (or even that there was a decrease although that is arguably less likely). Several of the examples from the contradiction papers may be better represented as a comparison claim [28–31] that uses a ternary relationship (rather than the binary relationship in an explicit claim) that captures at least two entities that are being compared, and the measure that was used in the comparison. For example, *cd-induced apoptosis* was used to compare the cells in the gradable comparison sentence ‘[Cd-induced apoptosis]_outcome_ was higher_gradable_ in [GSK-3beta-knockdown cells]_entity1_ than in [normal cells]_entity2_.

The claims reported here are also similar to manually constructed networks that capture statistically significant relationships between gene and proteins and cell proliferation [32] or cell death [33]. In contrast to that work, we do not constrain the relationships to only those that are statistically significant. The results for the 27 chemicals analyzed here show a high level of disagreement reported in the literature that can be seen clearly in the waffle plots but would be very difficult to discern from a dense ‘hair ball’ network graph.

Both entity and argumentation efforts have discussed elliptical coordinated compound noun phrases (CCNPs), where an author will save space by not repeating words, for example, an author will use the backward ellipses *T or B cells* rather than *T cells or B cells*. Without dealing with CCNPs, the system would fail to capture T cells. Annotators who constructed the GENIA corpus could mark the non-consecutive text, but most argumentation systems do not deal with this phenomenon. For example, in [26], *liver and cardiac toxicities* was marked as 1 entity which resulted in low inter-annotator agreement because some entities in lists such as eanthralogia/myalgia were separated, but CCNPs were not. In [25], the coordination issue was avoided by asking annotator to identify the entire sentence that supported or contradicted a query. For example, annotated the sentence “Among older adults, consumption of tuna or other broiled or baked fish, but not fried fish is associated with lower incidence of CHF’ was identified as causal, but in the claim framework this would be characterized as an association (not an explicit aka causal claim) and broken into 4 separate associations between tuna and CHF, broiled fish and CHF, baked fish and CHF and the negated association between fried fish and CHF. We process CCNPs using the approach in [10], where syntax from the Stanford Dependency grammar [34] is used to identify candidate forward (e.g. cell death and proliferation), backward (e.g. T and B cells) and complex (e.g. normal human and animal cells) ellipses. A semantic strategy is then employed that uses rules (e.g. if a word appears multiple times when expanded the candidate phrases is not included) and heuristics (the number of times that a modifier is used with a head noun) to establish which noun phrases should be expanded. Experiments with 21,280 full text articles showed that more than 1 million noun phrases were impacted by coordinated ellipses and that 10.79% of all noun phrases would be missed if coordination were not addressed. The approach achieved 73% precision, 75% recall, 74% F-score and 95% accuracy for new noun phrases. Precision was higher for backward (82.62 vs. 78.63) and forward expansions (64.82 vs. 60.17) coordinated noun phrases and lower for complex expansions (63.41 vs. 72.59).

## Materials and methods

### Search strategy

**Fig 1:**
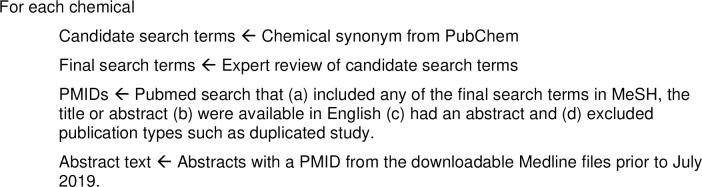
Search process used to identify MEDLINE abstracts for each of the 27 chemicals (see S1 Appendix for the complete search strings).

The paper captures evidence about cell changes, death and proliferation associated with 27 chemicals with known genotoxic or non-genotoxic mode of actions [5] (see Table 4). Each chemical name along with the synonyms produced by PubChem were reviewed by an expert (JS) who searched for synonyms and reviewed the published literature in PubMed, references listed in identified manuscripts, and textbooks to ensure that the terms in PubChem were relevant. The PubMed search was conducted in July 2019 (see Fig 1 for additional constraints and S1 Appendix for the actual search strings used).

### Text preprocessing

The XML PubMed Baseline Repository (updated December 2018) were processed on an AWS server along with daily update files to July 14^th^, 2019 (the last file was pubmed19n1318). Markup tags in the XML were used to identify the Background, Objectives, Methods, Results and Conclusions sections from structured abstracts and any remaining markup tags were removed using JSoup [35]. Abstracts available in English were processed using a the Ling Pipe biomedical text class [36] with additional abbreviations that occur frequently in the biomedical literature. After processing, non-ASCII characters in the extended ASCII set were replaced with ASCII approximations such as removing tildes and carons. MEDLINE abstracts can include itemized lists such as “Four categories represented a positive correlation: (1) increasing abnormal CEA with progressing disease, (2) decreasing abnormal CEA with disease regression, (3) unchanged abnormal CEA with stable disease, (4) change from normal to abnormal CEA with progressive disease.”(PMID 982100) that can interfere with the dependency parse. Thus, sentences were further processed to convert lists depicted with (a), (b) and (1), (2) into the constituent parts in order to improve the quality of subsequent parsing. In the example above the preamble of “Four categories represented a positive correlation” would be the first sentence and each of the constituent list items would become a separate sentence. Lastly, dependencies were generated using the Stanford parser version 1.9.2 [34].

During the preprocessing the system identifies and resolves elliptical coordinated noun phrases using the process described in [10] (see related work). For example, sentence 4 mentions two cells *p53-effective cells* and *p53-defective cells*, however the word “*cells”* appears only with the second of these noun phrases. Without attending to elliptical coordinated noun phrases, the system would detect that p53-defective cells had been induced but would neglect to capture that p53-effective cells were also induced.

4 Example sentence with coordinated ellipses: The p53 transactivation target Gadd45alpha was induced in both p53-effective and p53-defective cells after 4 h cadmium treatment, and this was associated with an acute inhibition of mitosis. (PMID 17174997)
  - p53-effective cells        b. p53-defective cells

Of the 482,101 abstracts retrieved using the search strategy only 76,587 (15.89%) provide section headings and of the structured abstracts most (69,901, 91.27%) include a result or conclusion section. BioBERT embeddings were used, which is a pre-trained Bidirectional Encoder Representations from Transformers (BERT) [37] model that was trained using biomedical text [38]. The model was trained on the structured abstracts to predict result or conclusion sentences in the unstructured abstracts. The model performed well on structured abstracts (accuracy 0.9363, F1 0.9396, precision 0.9464, recall 0.9329) and on a set of 560 manually annotated unstructured abstracts comprising 4,793 sentences that were assessed by 3 annotators (accuracy 0.9404, F1 0.9561, precision 0.9525, and recall 0.9597).

### Target outcomes

We introduce a semantics approach that combines knowledge resources and human natural language processing to overcome surface level differences between the way that knowledge is represented in a formal ontology and how authors discuss those concepts in abstracts.

**Fig 2:**
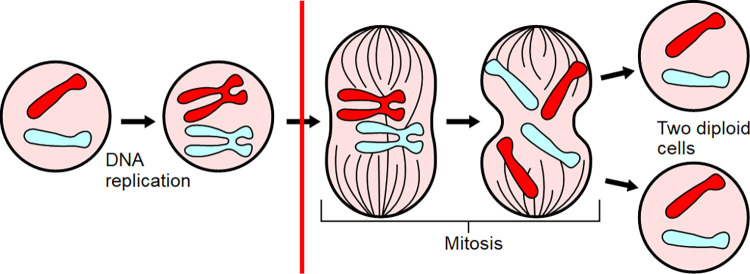
Cell proliferation outcome [41].

In this paper, two primary outcomes cell proliferation and death capture critical points in the cell life cycle and the underlying mechanisms associated with cancer. A broad definition of proliferation would include explicit mentions of cell proliferation along with any genes, processes, biomarkers, and assays [39] that are involved in the process. Although genes are common markers of cell proliferation [40], the National Cancer Institute’s definition of cell proliferation is used in this paper, which is “An increase in the number of cells as a result of cell growth and cell division” (see Fig 2). Thus, the mitosis step of the cell life cycle is within scope, but changes within the cell such as DNA replication is out of scope (DNA replication is also considered separately in [5]), as are changes in enzymes (most notably changes in peroxisome proliferation) and tumor changes that do not refer to cells. Abstracts that include proliferation indexes and mitotic markers are also detected and included as a cell proliferation target outcome. The second target outcome of cell death includes direct mentions of cell death along with necrotic and apoptotic expressions. The secondary outcome in this study captures any mention of cell changes that are not cell death or cell proliferation. This is essentially a less specific reference to the target primary outcomes.

**Fig 3:**
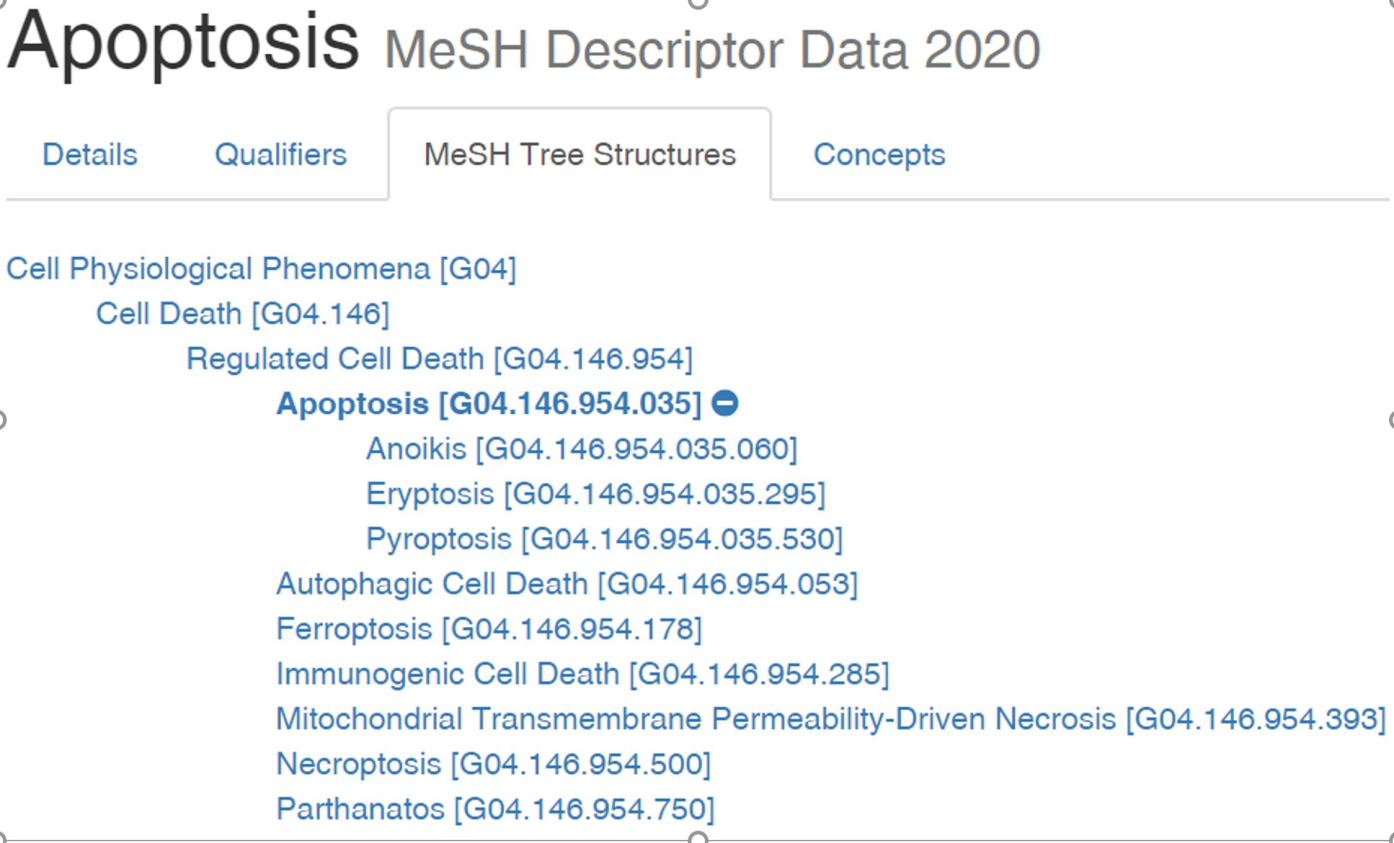
Partial view of the unified medical language system showing the Medical Subject Heading (MeSH) showing that *apoptosis* is a type of *Regulated Cell Death*, which is in turn a type of *Cell Death*. Image courtesy of the U.S. National Library of Medicine.

The semantics approach we propose combines knowledge resources with a natural language processing method that tends to ellipses. First expressions for the target concepts–in this paper cell, cell death, and cell proliferation are drawn from the Unified Medical Language System (UMLS) and online thesauri. The UMLS organizes knowledge as concepts (identified using a Concept Unique Identifier (CUI)) that unifies expressions from hundreds of different medical ontologies to improve the system recall (i.e. so that entities of interest are not missed). For example, the CUI for *Cell Death* is C0007587 and includes *apoptosis* in which cells are no longer needed and *necrosis* where the cells die due to injury. The MeSH taxonomy (one of the resources within the UMLS) includes the more general concept of *Regulated Cell Death* and the narrower concept of *anoikis*, a form of programmed cell death (see Fig 3). Thus, a specific ontology (in this case MeSH), can enable a user to crisply define the scope of their target outcome measures. Fig 3 shows only MeSH, but the UMLS online browser (https://uts.nlm.nih.gov/uts/umls/home) which was used to identify the expressions in this paper includes multiple ontology and thesauri resources.

**Fig 4:**
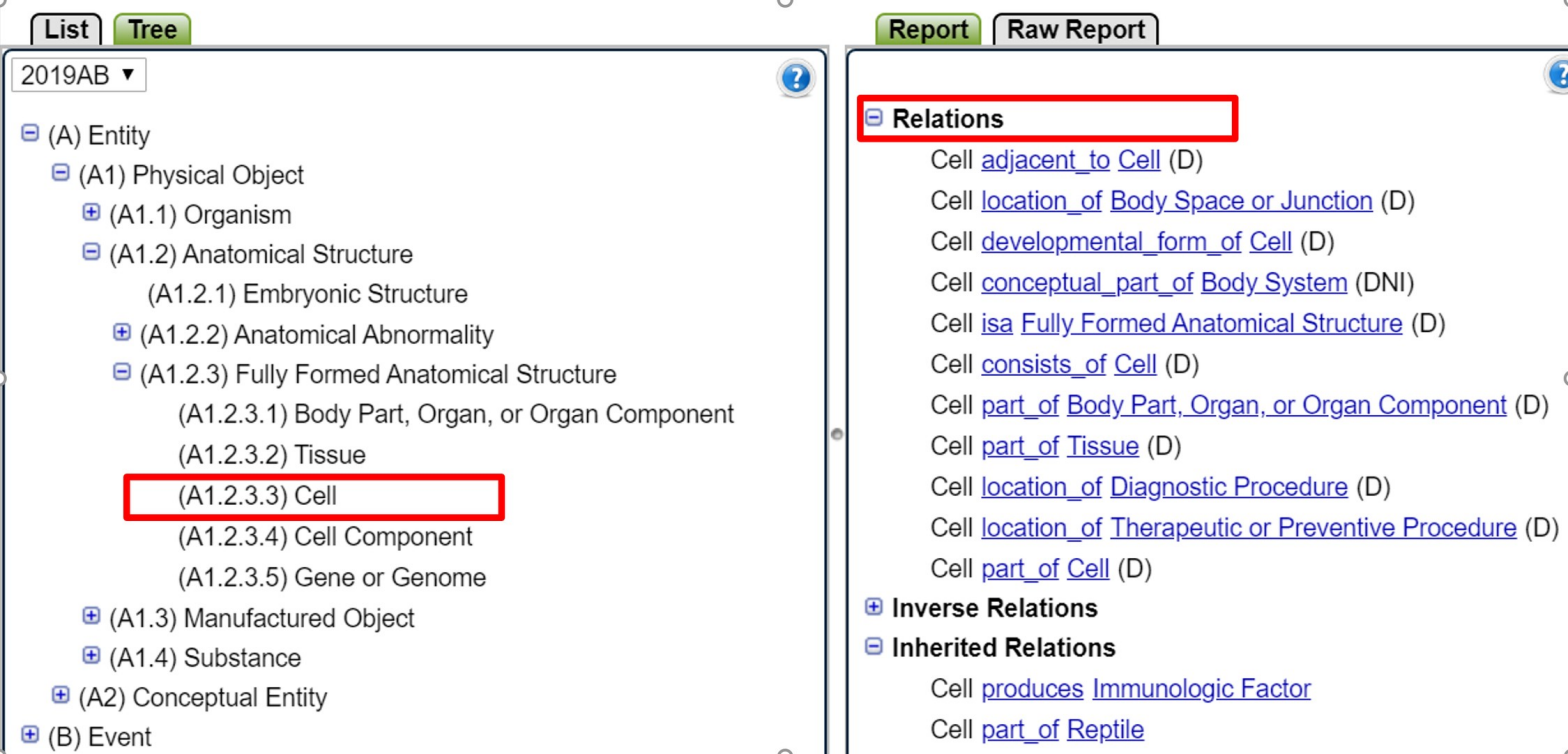
Unified Medical Language System (UMLS) showing context for the semantic type *cell*. Image courtesy of the U.S. National Library of Medicine.

Each CUI in the UMLS is assigned 1 or more of the 134 semantic types that capture categories of concepts that can identify additional concepts that are broader or narrower than the initial target outcome. Cell is a both concept name and a semantic type (see Fig 4 which shows Cell as a semantic type). As with concepts, the UMLS enables a user to add additional expressions by exploring more general semantic types such as *Fully Formed Anatomical structures* to sharply define the target outcome and ensure good coverage.

In a knowledge-based approach, terms from the knowledge resource are compared directly with text in the abstract. However, the scientific literature often includes modifiers that are not mentioned explicitly in an ontology and surface level differences such as coordinated ellipses mean that an exact match strategy would miss target outcome expressions. Two strategies were used to overcome this issue. First, natural language processing is used to attend to coordinated ellipses (see text preprocessing). Second, the primary target outcomes were characterized as either single or multi-word expression. Any phrases containing a single word expression such as *angioproliferate* and *apoptosis* were included in the set of target outcomes. Multi-word expressions were deconstructed into <cell> <proliferation> and <cell><death> and then combinations of words capturing synonyms of cell, proliferation, and death were combined to form the target outcomes. The UMLS was searched using the online UMLS browser, and online dictionaries and thesaurus were consulted. All terms were verified by the domain expert (JF) who augmented her expertise with searches in PubMed, reference reviews and textbooks to produce a dictionary of cell terms. Very few additional terms were added during the manual step of this process. Similarly, a set of synonyms for proliferation and death were identified. Lastly phrases that included at least 1 cell term and either 1 proliferation or 1 death term were included as target outcomes. Phrases that included ’pathway’ or ’pathways’, or a word that started with ’factor%’ or ‘’peroxisom%’ were removed to satisfy the exclusion criteria and avoid including tumor necrosis factor (TNF) and phrases involving peroxisomes.

In addition to noun phrases, the primary target outcome entities can be expressed as a prepositional phrase, such as *proliferation of cultured gastric cancer cells* which is illustrated in sentence 5. For these cases, the claim framework was used to detect changes where an increase in cells was captured as cell proliferation and a decrease in cells was captured as cell death.

5 Example sentence where the outcome is a prepositional phrase: Enzastaurin suppressed the proliferation of cultured gastric cancer cells and the growth of gastric carcinoma xenografts. (PMID 18339873)

### Extracting claims

The Claim Framework captures how scientists communicate results and comprises five types of claims: explicit, implicit, comparisons, associations, and observations [11]. An explicit claim is the most frequent claim type used in full text articles and requires two entities (an entity that has been changed and an entity that is responsible for the change), along with a change term that captures how the first entity changes the second. The analysis reported here considers only the entity that has been changed where entity is constrained to cell death, cell proliferation, and cells changes that are not death or proliferation. An observation claim reports how an entity has changed but does provide information about what was responsible for the change in the same sentence, such as in, ‘Results show a statistically significant increase in cell proliferation’.

Both semantics and syntax are used to identify claims automatically. The semantics in the initial system to detect explicit claims [11] uses a set of anchor terms comprising 174 directionality verbs (55 indicating an increase and 74 indicating a decrease) and 208 change verbs from the TREC collection [42]. An evaluation using abstracts from [5] resulted in updates to the initial system, which now uses 215 directionality terms (58 increasing, 86 decreasing, and 71 general change verbs and 235 causality verbs). As with the initial version, the base form of each verb is expanded to capture all tenses and nominalized forms before being comparing with the abstract text. Observational claims, where an author does not specify the entity responsible for the change, had not been previously implemented. The system now detects observations using the same syntax and semantics for the explicit claims, but where the entity responsible for the change is not identified. The preprocessing step that reconciles coordinated ellipses has also been added to the system so that example the neutral change to human lymphocyte proliferation is captured from “Inorganic arsenic effects on human lymphocyte stimulation and proliferation” and the negated neutral change on proliferation and apoptosis is captured from “It had no effect on proliferation, apoptosis, or differentiation”.

With respect to syntax, a set of rules were constructed that connect each anchor term through dependency paths from the Stanford parser to the target outcomes. In addition to a direct connection between an anchor and a target outcome (e.g. increases cell death), the system captures connections through a prepositional phrase (e.g. induction of apoptosis), and through measurement terms (e.g. the *amount of* cell death).

The error analysis revealed that one last change was needed to the original system because the target outcomes in this paper implicitly indicate a change, such as in sentence 6, where cell proliferation is captured a prepositional phrase. To resolve this issue explicit claims and observations were first applied to the text. The syntactic rules were then reapplied to any increase in cells for cell proliferation and any decrease in cells for cell death. Thus, the system would report a refuting cell proliferation for sentence 6, where inhibit is the change term and *proliferation of U251 malignant glioma cells* captures the target outcome cell proliferation.

6 Example sentence showing where the syntactic rules were applied twice: Released irinotecan inhibited the proliferation of U251 malignant glioma cells. (PMID 24460101)

In a manual systematic review, an extraction worksheet helps reviewers identify the results of a study [1] and our system is strongly influenced by this human practice. To avoid capturing claims that reflect an author’s description of previous work or their proposed hypothesis that has yet to be verified, the system only includes sentences from the result or conclusion section, where the section is labeled as result or conclusion in structured abstracts and where the label is predicted from a deep learning model for unstructured abstracts (see text preprocessing for details).

### Characterizing evidence

Supporting evidence is either an explicit or observational claim where the target outcome has increased. Refuting evidence reports a decrease in the target outcome and neutral evidence shows that there is a change, but the language used in the abstract lacks the specificity to determine if the target outcome has increased or decreased. Negation is also captured and can occur within the noun phrase or within the relation, thus there are 12 possible claim directions. Table 1 shows examples for the target outcome cell proliferation and includes both noun phrase and non-noun representations.

**Table 1:** Cell proliferation example showing how claims and outcomes are aligned with the direction of evidence.

Claim		Out-come	Noun Phrase	Non-Noun Phrase	Con-clusion
	^		**… increased** cell proliferation … (11852482)	… **induced** proliferation of mammary epithelial cells. (10965359)	Support
	-		… the short-term **effects** of various agents on cell proliferation … (17875779)	… **effects** promoting cell growth … (21771884)	Neutral
	v		… directly **inhibit** DLD-1 cell growth … (21098876)	… **down-regulates** proliferation of choriocarcinoma cells (11352660)	Refute
¬	^		Cell proliferation was ***not*** inhibited by … (18296742)	… ***failed*** to **stimulate** the growth of WY-1 and WY-20 cells … (1740016)	Negated Support
¬	-		… had ***no* effects** on cell proliferation. (18395325)	… had ***no*** apparent **influence** on the proliferation of PML/RARalpha-positive stem cells … (17339181)	Negated Neutral
¬	v		… did ***not* inhibit** cell growth. (17415774)	Growth of HepG2 cells in culture was ***not* inhibited** by … (20377131)	Negated Refute
	^	¬	… MPP(+)-**induced** sympathetic neuron loss … (20079841)	… **contributes** to the loss of such neurons … (21975039)	Refute
	-	¬	A strong synergistic **effect** on antiproliferative and proapoptotic activity was found… (21390185)	… **effect** on loss of thymocytes is … (12032332)	Neutral
	V	¬	… **reduction** in striatal and cortical cell loss (20976216)	… to **diminish** bulbectomy-induced loss of NRD neurons … (9075262)	Support
¬	^	¬	PQ did ***not* induce** neuronal cell loss or… (27788145)	… did ***not* induce** the loss of TH expression or DA neurons … (23500093)	Negated Refute
¬	-	¬	… had ***no*** appreciable **effect** on its antiproliferative activity (12538494)	… had ***no* effect** on loss of cell viability … (14617789)	Negated Neutral
¬	V	¬	… did ***not* prevent** TH-positive cell loss. (16464239)	… ***not* prevent** the loss of conjunctival goblet cells … (31038554)	Negated Support

The first column captures the negation (¬) and the polarity (^ depicts support,—depicts neutral, and v depicts refuting evidence) of the relationship and the second column captures if the outcome is negated. PMIDs are shown in parenthesis.

An abstract can report multiple directions of evidence for the same target outcome and sometimes within the same sentence. Consider sentence 7 for the target outcome *necrotic death* where the system captures two directions of evidence from the words *triggered* that is supportive and *attenuates* that is refuting.

7 Sentence with multiple lines of evidence for the same outcome: We show here that Nec-1 also effectively attenuates necrotic death triggered by Cd. (PMID 19135076)
  - Supporting    triggered necrotic    death
  - Refuting    attenuates    necrotic death

The system first identifies outcomes (see section on target outcomes) and then identifies claims that include those outcomes; thus, negation can be applied to the claim, the outcome, both the claim and the outcome or neither the claim nor the outcome. Table 1 provides a summary of how negation at the claim level that also includes the polarity (support, neutral or refuting) and the entity level are reconciled to arrive at the direction of evidence that are reported and shown as visual summaries. The direction of evidence is ordered from left to right with respect to the extent to which the outcome supports a change in evidence i.e. (Refute -> Negated Refute -> Neutral ->Negated Neutral -> Negated Support ->Support).

It’s not clear if an abstract that reports the same claim multiple times should be considered more compelling than an abstract that makes a claim only once. Consider 3 example thiobenzamide abstracts that report changes in cell proliferation. As shown in Table 2 all three abstracts included 2 supporting claims, and the second abstract also has refuting and neutral claims. If the number of claims is considered then cell proliferation would have 1 refuting claim, 1 neutral claim, and 6 supporting claims (n = 8). However, if the number of abstracts were considered then there would be 1 refuting abstract, 1 neutral abstract and 3 supporting abstracts (n = 3).

**Table 2:** Claim vs abstract totals for 3 example thiobenzamide (chemical 27) abstracts.

ID	Claim Counts	Abstract Counts
1	Support	1 support for abstract 1
1	Support	
2	Support	1 support for abstract 2
2	Support	
2	Refute	1 refute for abstract 2
2	Neutral	1 neutral for abstract 2
3	Support	1 support for abstract 3
3	Support	

## Results and discussion

### Target outcome detection

There are no gold standards that capture the directionality of cellular outcomes, cell death, and cell proliferation. An earlier study used machine learning to detect cell death and cell proliferation abstracts during the retrieval stage of a risk assessment. The accompanying manual annotations had substantial agreement (Kappa statistic 0.68) for inter-rater reliability [4]. We require that authors explicitly mention cell proliferation or cell death (or a synonym) and many of the 340 abstracts that were annotated as cell proliferation (out of 3,078 total abstracts from 15 journals), or cell death (380 abstracts) do not mention the target outcomes. The annotations from the earlier work suggests that annotators were inferring cell proliferation from internal cell processes such as peroxisome proliferation, which does not always lead to cell proliferation. We include mitogens, a protein that induces a cell to proliferate, but it appears that the previous work did not identify those abstracts. It is not clear if the annotators in the prior work were asked to identify all abstracts that measure cell proliferation or death, or if they were only asked to identify abstracts in which these outcomes increased. These nuances underscore the need to provide a clear definition of each target outcome as part of the system reporting and clear instructions that identify any abstract where the target outcome was measured, regardless of the result.

**Fig 5:**
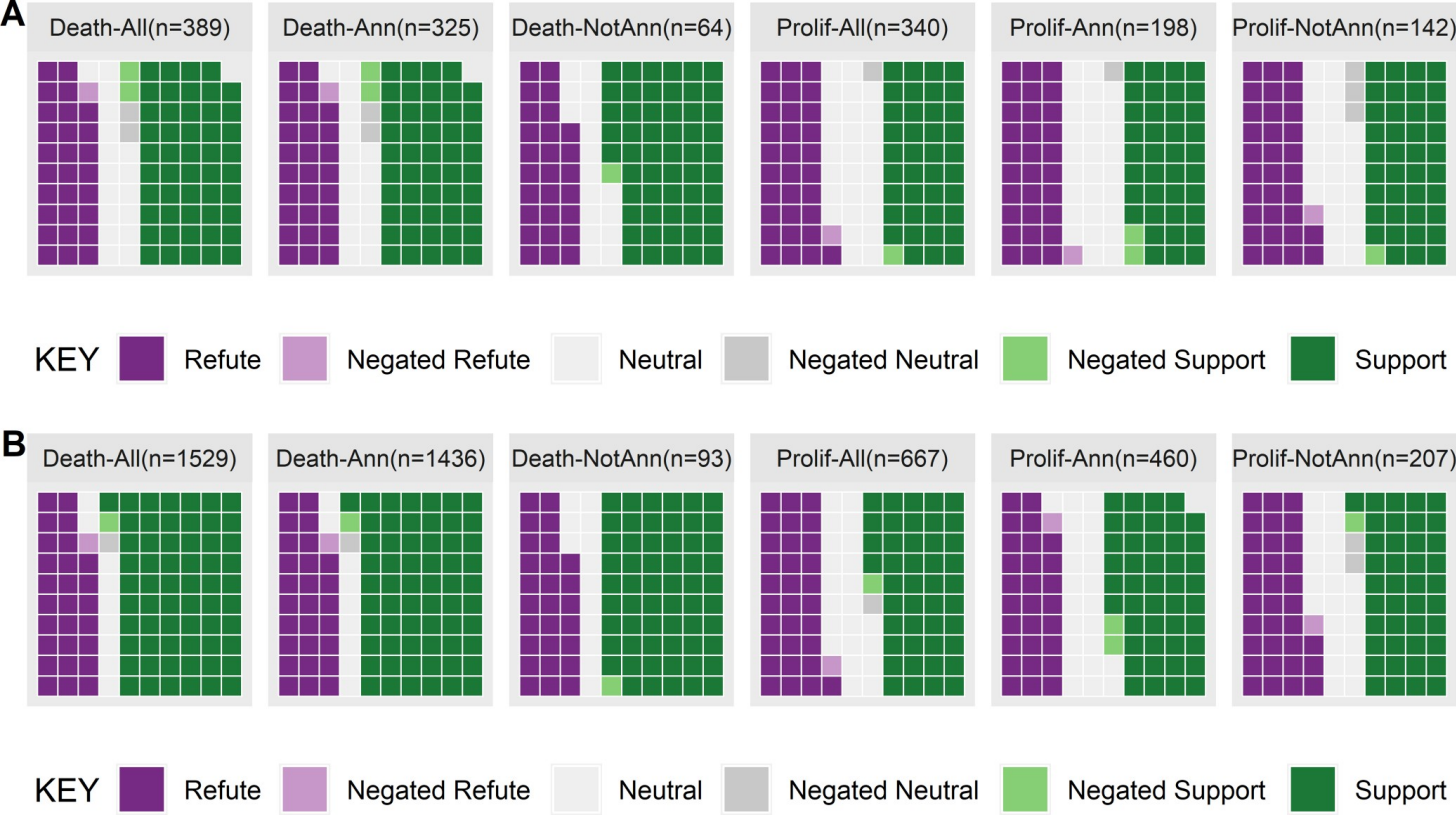
Claims concerning cell proliferation and cell death reported in [4] showing the entire collection (All), abstracts that were marked as relevant (Ann) and abstracts that were not manually annotated as relevant (NotAnn) from [4], where 5A shows abstract frequencies and 5B shows claim frequencies.

Differences in the scope limit the utility of measuring precision and recall of our system with respect to the earlier manual annotations so we focus instead on how the different scoping choices might change the subsequent decision making. Specifically, is there a difference between claims in the entire collection (i.e. reported anywhere in the 3078 abstracts), compared with claims made in the annotated abstracts and un-annotated abstracts? For cell death, the abstracts that were not in the manually identified set of abstracts had a greater proportion of supportive evidence than those in the manually annotated abstracts (see Fig 5). In contrast, for cell proliferation, abstracts that were not manually annotated but did report cell proliferation had a greater proportion of refuting evidence. This is consistent with the distribution of evidence found in our larger collection of 482,314 abstracts.

### Outcome mentions

Human language often follows a power law distribution where a small number of expressions capture a large proportion of the expressions; thus, the target outcomes were evaluated by manually inspecting the 100 most frequent expressions for each of the primary target outcomes. There were 7 errors for cell proliferation, a reference to an assay, an increase in cell size rather than the number of cells, mitotic spindle, 2 expressions for cell migration, an antiproliferation agent, and geo-accumulation index. There were 14 errors for cell death, 8 expressions referred to proteins, 3 referred to genes, an apoptosis assay, apoptotic potential and apoptotic mechanism.

The top 20 terms (see Table 3) show that authors are more likely to use negation with cell proliferation, where the 4^th^ and 5^th^ most frequent expressions capture an antiproliferative effect or activity, but there is only 1 negated cell death term in the top 20. There were 24,435 cell proliferation expressions, 16,591 cell death expressions, and 195,903 cell mentions that were neither proliferation nor death. This suggests that the approach is robust with respect to additional modifiers that were not in the original knowledge base.

**Table 3:** Twenty most frequent lemmatized outcome expressions for cell proliferation and cell death, where * depicts negation and depicts a non-noun phrase.

Cell Proliferation		Abs	Freq	Cell Death		Abs	Freq
	cell proliferation	4,755	6,482		apoptosis	11,451	23,132
	cell growth	2,114	2,576		cell death	3,852	5,545
	increase_cell	1,057	1,120		necrosis	1,599	2,122
*	antiproliferative effect	797	983		cell apoptosis	1,200	1,438
*	antiproliferative activity	700	949		cell cycle arrest	901	1,124
	proliferate_cell	620	690		apoptotic cell death	842	1,019
	proliferate_cell nuclear antigen	589	705		apoptotic cell	819	1,081
	cellular proliferation	589	685		tumor necrosis	813	864
	lymphocyte proliferation	274	354		program cell death	475	588
	mitotic index	266	352		apoptotic effect	333	395
	tumor cell proliferation	247	284		apoptosis induction	271	308
*	loss_dopaminergic neuron	235	264	*	antiapoptotic effect	237	310
	t cell proliferation	207	285		neuronal cell death	235	298
	cellular accumulation	187	224		apoptotic	233	249
	increase_cell viability	177	184		arrest_cell cycle	220	235
*	loss_cell viability	166	182		proapoptotic effect	219	248
	growth_cell	163	170		arrest_cell	210	235
	hepatocyte proliferation	162	239		kill_cell	208	227
*	cell loss	153	176		neuronal apoptosis	197	278
*	cell growth inhibition	145	161		acute tubular necrosis	190	240

With respect to ellipses, 5,402 cell proliferation expressions from 4,283 abstracts would have been missed if ellipses were not resolved (the most frequent expressions were cell proliferation, antiproliferative effect, cell growth and antiproliferative activity). With respect to cell death, 5,880 expressions from 4,566 abstracts would have been missed (the most frequent expressions were cell apoptosis, cell cycle apoptosis, oxidative apoptosis, and growth apoptosis) if the system did not resolve coordination. For general cell terms, 46,823 terms from 29,113 abstracts were added (frequent expressions were cell differentiation, cell migration, cell invasion, and normal cell).

Table 4 shows that the search criterion identified 482,314 abstracts relevant to the 27 chemicals, where the number of abstracts ranged from 118 for Thiobenzamide (chemical 27) and 186,580 for Pyridine (chemical 23). With respect to the target outcomes, cell proliferation was reported more often than cell death (average 7.5%, min 3.2% and max 32.6% versus average 5.6%, min 1.1% max 26.5%) and general cell terms were reported in 36.1% of the abstracts (min 15.8% and max 86.7%).

**Table 4:** Total target outcome mentions in the collection (regardless of directionality).

			Abstracts that mention an outcome												Total outcome mentions								
			Anywhere						Result or Conclusion Section						Anywhere			Result or Conclusion Section					
ID	Chemical Name	Total	Prolif	%	Death	%	Cell	%	Prolif	%	Death	%	Cell	%	Prolif	Death	Cell	Prolif	%	Death	%	Cell	%
1	1,3-Butadiene	3,493	136	3.9	93	2.7	595	17.0	104	76.5	79	84.9	455	76.5	243	297	2,507	168	69.1	196	66.0	1,364	54.4
2	4-Aminobiphenyl	770	35	4.5	10	1.3	252	32.7	27	77.1	10	100.0	183	72.6	56	17	848	39	69.6	16	94.1	455	53.7
3	5-Azacytidine	6,501	1,403	21.6	902	13.9	5,638	86.7	1,091	77.8	737	81.7	4,808	85.3	2,330	2,326	33,846	1,554	66.7	1,480	63.6	18,625	55.0
4	Arsenic	22,496	1,511	6.7	1,933	8.6	6,235	27.7	1,211	80.1	1,699	87.9	5,075	81.4	2,825	6,910	29,161	1,928	68.2	4,315	62.4	16,372	56.1
5	Asbestos	7,780	672	8.6	332	4.3	2,851	36.6	550	81.8	264	79.5	2,289	80.3	1,210	877	13,339	846	69.9	556	63.4	7,612	57.1
6	Benzo-a-pyrene	12,160	988	8.1	585	4.8	5,382	44.3	827	83.7	516	88.2	4,353	80.9	1,727	1,748	27,239	1,249	72.3	1,187	67.9	15,612	57.3
7	Bisphenol A	12,946	829	6.4	416	3.2	3,075	23.8	680	82.0	351	84.4	2,422	78.8	1,610	1,015	13,981	1,087	67.5	676	66.6	7,534	53.9
8	Cadmium	40,300	1,711	4.2	1,717	4.3	11,751	29.2	1,426	83.3	1,487	86.6	9,652	82.1	2,681	5,455	49,993	1,989	74.2	3,578	65.6	29,794	59.6
9	Chloroform	18,987	667	3.5	488	2.6	4,500	23.7	522	78.3	412	84.4	3,432	76.3	1,427	1,281	15,273	840	58.9	846	66.0	8,892	58.2
10	Cyclosporine	37,290	4,090	11.0	3,309	8.9	18,685	50.1	3,085	75.4	2,644	79.9	14,450	77.3	7,226	11,172	90,912	4,774	66.1	6,749	60.4	49,422	54.4
11	Dichloroacetate	986	152	15.4	183	18.6	469	47.6	120	78.9	143	78.1	396	84.4	279	472	2,512	187	67.0	274	58.1	1,309	52.1
12	Diethylnitrosamine	4,233	1,174	27.7	530	12.5	2,884	68.1	920	78.4	455	85.8	2,228	77.3	2,330	1,286	12,275	1,465	62.9	882	68.6	6,816	55.5
13	Diethylstilbestrol	5,596	671	12.0	202	3.6	2,524	45.1	534	79.6	168	83.2	2,067	81.9	1,354	593	11,643	917	67.7	381	64.2	7,107	61.0
14	Ethene Oxide	6,857	227	3.3	76	1.1	1,400	20.4	179	78.9	60	78.9	1,056	75.4	348	155	4,751	241	69.3	95	61.3	2,606	54.9
15	Formaldehyde	50,707	3,692	7.3	2,211	4.4	22,941	45.2	2,540	68.8	1,571	71.1	17,487	76.2	6,675	5,391	101,063	3,730	55.9	2,918	54.1	58,836	58.2
16	Fumonisin_B1	1,858	242	13.0	430	23.1	854	46.0	187	77.3	381	88.6	721	84.4	489	1,827	4,169	315	64.4	1,147	62.8	2,403	57.6
17	Genistein	10,383	2,180	21.0	1,219	11.7	7,149	68.9	1,674	76.8	1,039	85.2	6,088	85.2	4,724	4,416	39,064	2,894	61.3	2,776	62.9	21,341	54.6
18	Irinotecan	7,057	615	8.7	742	10.5	2,696	38.2	454	73.8	602	81.1	1,883	69.8	1,026	2,054	11,644	648	63.2	1,272	61.9	6,055	52.0
19	Methylene Chloride	10,861	343	3.2	237	2.2	1,720	15.8	272	79.3	210	88.6	1,295	75.3	657	737	5,619	394	60.0	462	62.7	3,071	54.7
20	Nafenopin	261	85	32.6	50	19.2	179	68.6	67	78.8	47	94.0	159	88.8	218	356	914	125	57.3	232	65.2	583	63.8
21	Okadaic Acid	5,430	827	15.2	767	14.1	4,208	77.5	617	74.6	611	79.7	3,655	86.9	1,552	2,925	21,648	999	64.4	1,771	60.5	12,890	59.5
22	Phenobarbital	16,667	845	5.1	547	3.3	4,089	24.5	684	80.9	458	83.7	3,219	78.7	1,641	1,435	16,635	1,113	67.8	990	69.0	9,927	59.7
23	Pyridine	186,580	12,363	6.6	9,592	5.1	60,982	32.7	9,517	77.0	7,814	81.5	47,604	78.1	21,634	27,705	256,284	14,196	65.6	17,242	62.2	140,394	54.8
24	Styrene	16,780	719	4.3	280	1.7	3,872	23.1	568	79.0	226	80.7	3,113	80.4	1,222	659	18,704	819	67.0	406	61.6	10,040	53.7
25	Sulindac	1,444	300	20.8	383	26.5	703	48.7	240	80.0	325	84.9	594	84.5	669	1,490	3,453	420	62.8	865	58.1	1,975	57.2
26	TCDD	11,621	897	7.7	471	4.1	4,481	38.6	734	81.8	397	84.3	3,676	82.0	1,668	1,398	21,240	1,154	69.2	892	63.8	12,442	58.6
27	Thiobenzamide	118	7	5.9	13	11.0	24	20.3	7	100.0	9	69.2	20	83.3	13	21	61	11	84.6	16	76.2	43	70.5
		482,314	36,106	7.5	26,900	5.6	174,110	36.1	27,810	77.0	22,020	81.9	137,550	79.0	65,393	81,346	781,475	42,450	64.9	50,462	62.0	438,139	56.1

When conducting a risk assessment, the manual processes should only consider the results from the current study being reviewed, and not use an author’s interpretation of previous work. The system therefore should identify only the outcomes in the result or conclusion sections of an abstract. The total number of abstracts that include a result or conclusion target outcome was 27,810 for cell proliferation, 22,020 for cell death and 137,550 for a general cell term (see Table 4). Table 4 also shows the difference between the total number of outcomes mentioned anywhere in an abstract that would be identified during the retrieval step, and how many outcomes appear in the result or conclusion sentences. Table 4 also provides an approximate upper bound on the number of claims that can be identified within the collection (approximate because a single outcome can have multiple change terms). Note that some abstracts include more than one chemical.

### Claim extraction

To evaluate the precision of the claims extracted, a random sample of 50 sentences from each outcome were manually inspected. The accuracy was 81.3% (82% for cell proliferation, and 88% for cell death and 75% for general cells). Of the 150 sentences, 58 were refuting, 28 were neutral, and 64 were supporting and the accuracy was 84, 67 and 75% respectively which suggests that the system is more accurate with respect to refuting claims, than for neutral, or supporting claims.

With respect to recall, a random sample of 200 sentences (100 each for cell proliferation and death) were manually reviewed that did not capture a claim but included a primary outcome and at least 1 anchor term. Passive tense can be an issue for claim extraction so 50 sentences included an anchor term before the outcome, which are more likely to use active tense, and the 50 sentences used an anchor term after the outcome. There were 8 sentences that included a claim that was not detected (3 sentences before the outcome and 5 after) for cell proliferation and 13 sentences (7 before the outcome and 5 after) that missed a valid claim about cell death, producing a recall of 92% and 87% for proliferation and death respectively. It does not appear that passive tense impacts the recall of the claims.

**Fig 6:**
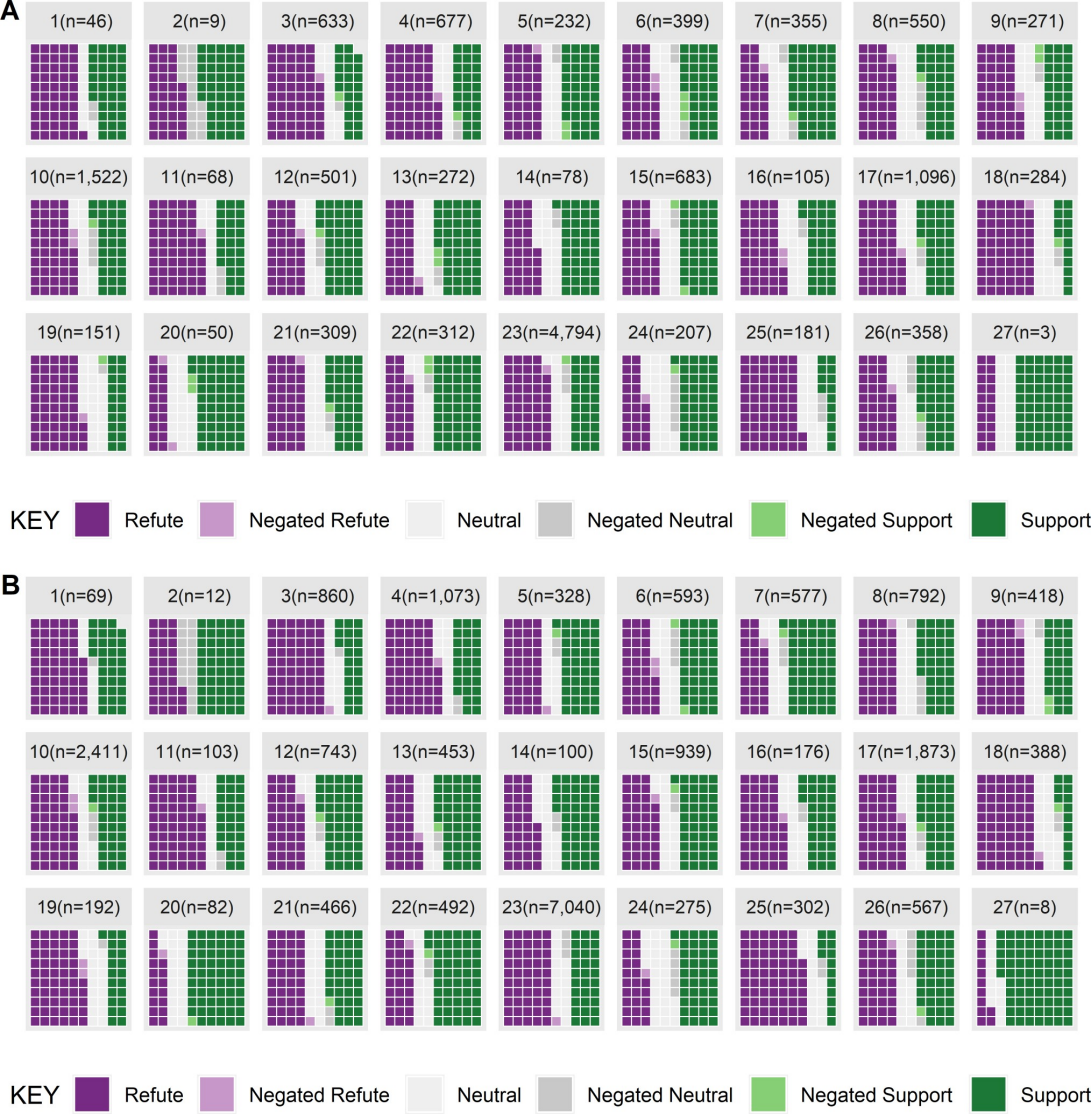
Cell proliferation claims from results or conclusions sentences showing [A] total abstracts and [B] total claims.

**Fig 7:**
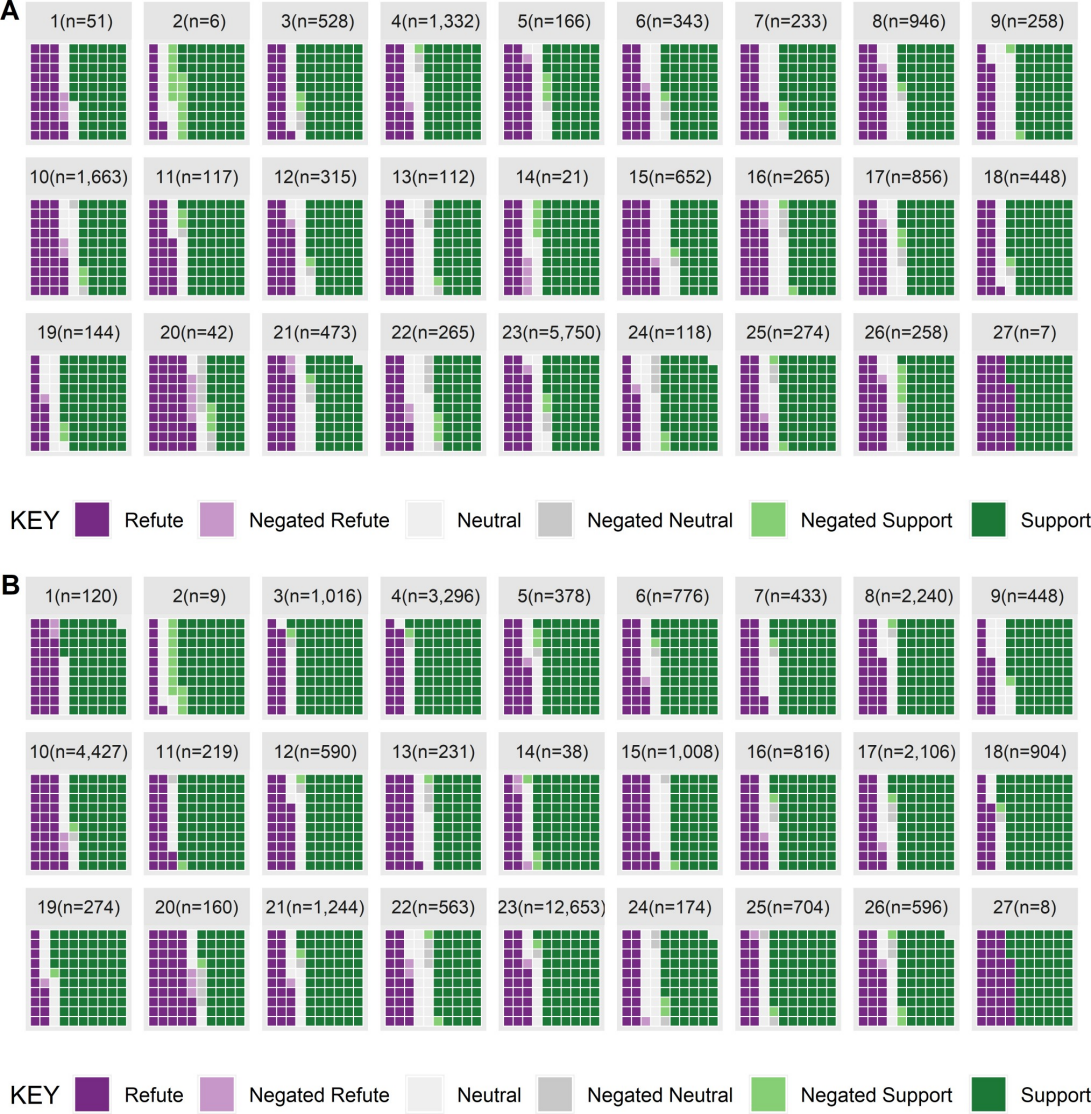
Cell death claims from results or conclusions sentences showing [A] total abstracts and [B] total claims.

**Fig 8:**
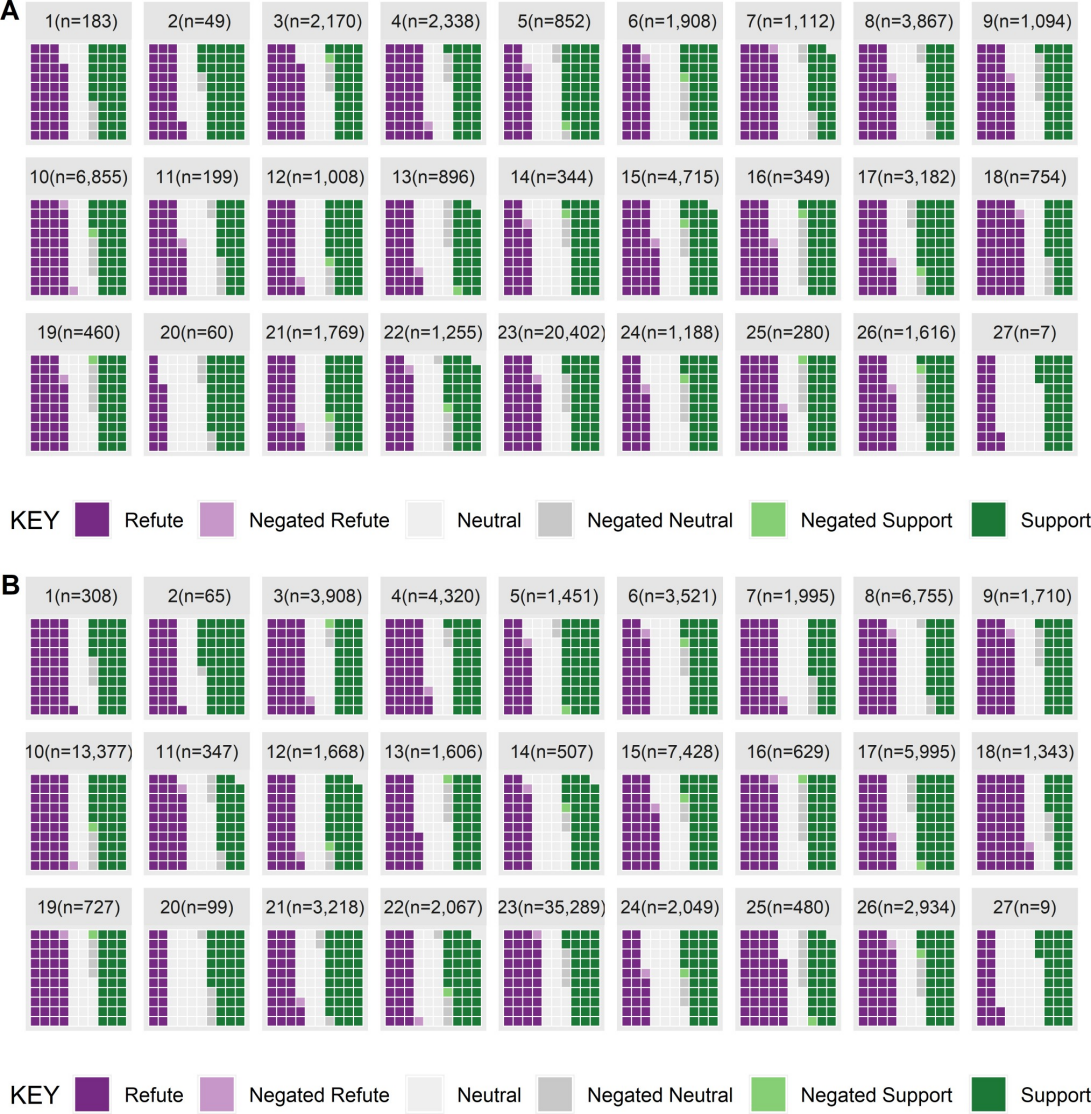
General cell claims from results or conclusions sentences showing [A] total abstracts and [B] total claims.

Table 4 shows the number of abstracts that report a target outcome and can thus be used during the retrieval step of a risk assessment, whereas Figs 6–8 show the distribution of refuting, neutral, and supporting evidence extracted. If all the abstracts (or claims) were supportive then the waffle plot would be entirely green. With respect to cell proliferation, 53.6% of abstracts included a refuting claim, 22% were neutral, and 38.7% were supporting (6.4% of abstracts included negated evidence). When considering the number of claims the rates are 45.9, 16.5 and 33% for refuting, neutral, and supporting claims respectively. None of the chemicals have entirely supporting evidence and more than half of the chemicals (15/27) have more refuting evidence than supporting evidence, such as Sulindac (chemical 25) where 67.2% of the claims refute the hypothesis that cell proliferation increases (see Fig 6). However, 12 chemicals do have more supporting evidence than refuting evidence with respect to cell proliferation.

In contrast to proliferation, 38.9% of abstracts refute the hypothesis that cell death increases, 19.7% provide neutral evidence, and 76.7% of the evidence is supportive (6.4% of the abstracts include negated claims). When considering the number of claims the rates are 25% refute, 10.6% neutral, and 61.3% support. None of the chemicals have more refuting evidence and 26 of the 27 chemicals have more supporting evidence than refuting evidence (see Fig 7).

The distribution between refuting, neutral, and supporting evidence for general cell changes were more evenly distributed than for cell proliferation or death and there were 46.6%, 34.5%, and 41.4% of abstracts (note that the total is greater than 100% as an abstract often reports more than 1 claim). When considering the number of claims, the distribution was 37.7%, 25.0% and 32.6% for claims that refute, were neutral, or supportive. (see Fig 8).

### Impact on decision-making

**Fig 9:**
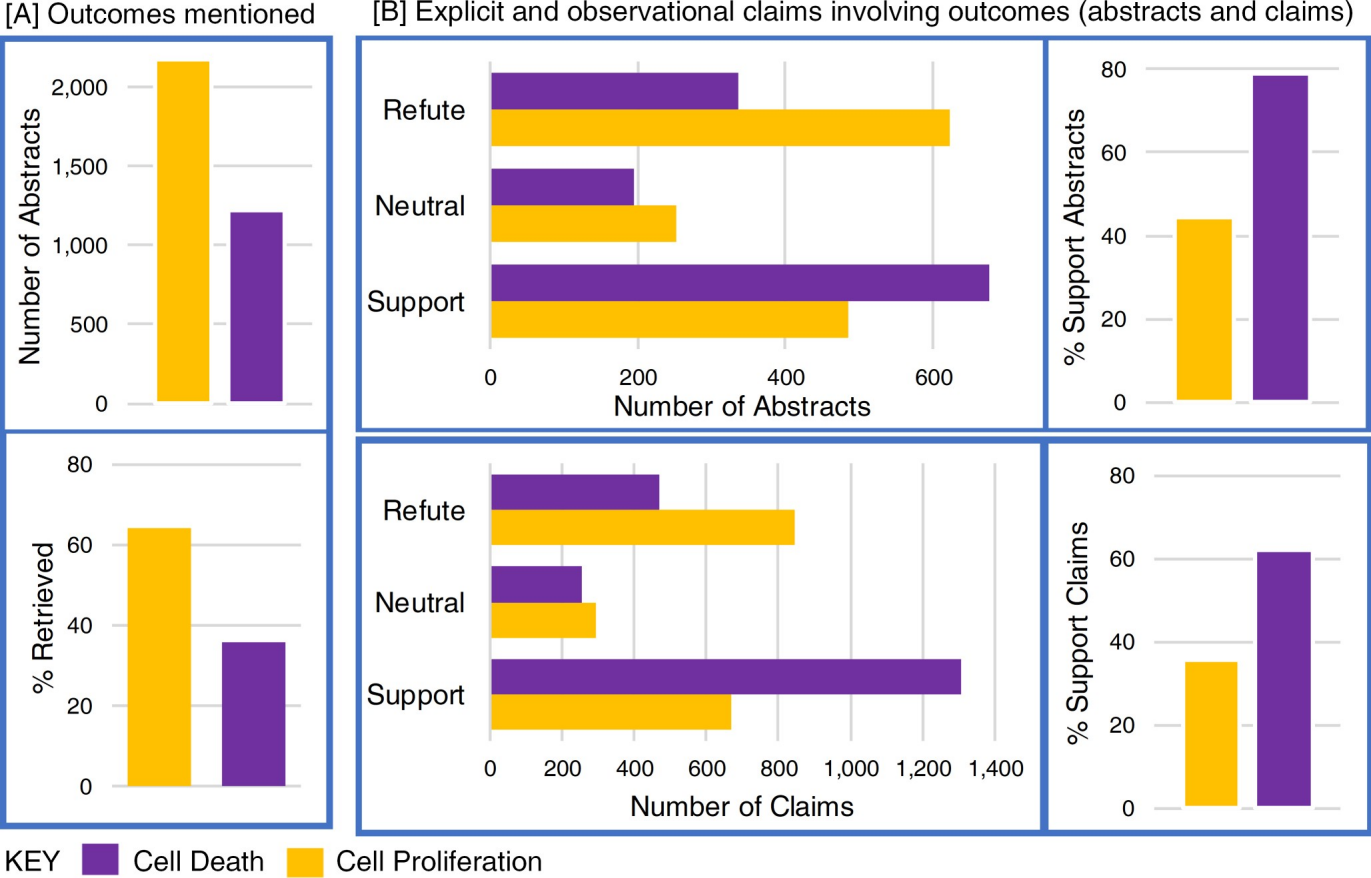
The number of genistein abstracts that report cell proliferation is greater than the number that report cell death (A); however, more claims refute that cell proliferation increases, whereas more claims support an increase in cell death.

Cell proliferation and death capture diametrically opposed biological processes within the cell cycle, so it makes sense to ask if a chemical is more strongly associated with cell proliferation or death and how that decision might change if using data from only the retrieval step, versus data from the extraction step that detects supporting, neutral, or refuting claims as shown in Figs 6–8. Consider, genistein (chemical 17), where more abstracts report cell proliferation than cell death (a finding that is consistent with [5]). The information retrieval step identifies abstracts that measure an outcome but measuring an outcome should not be confused with being associated with an outcome. Unfortunately, this distinction can be easily misinterpreted when presented with figures that capture the number of abstracts retrieved, as shown in Fig 9A (see Table 5, chemical 17 for the underlying data used in Fig 9). However, if the directionality of the claims is considered, then there are more abstracts that refute an increase in cell proliferation and more abstracts that support an increase in cell death (see Fig 9B). The result is the same if the number of claims (rather than number of abstracts) are considered, or if the percentage of supporting evidence rather than the raw numbers are considered (see Table 5). Thus, a decision maker would conclude that genistein is more closely associated with cell proliferation if considering only the abstracts retrieved, and cell death if considering the supporting evidence.

**Table 5:** Conclusion based on [A] the number of abstracts that mention the target outcome [B] the number of abstracts that include at least 1 claim where the target outcome has increased, and [C] the number of claims where the target outcome has increased. Data for [B] and [C] consider only the result or conclusion sentences (either actual or estimated).

[A] Retrieved abstracts				[B] Abstracts with > = 1 support claim						[C] Total support claims					
	Number			Number			%			Number			%		
ID	Prolif	Death	Concl	Prolif	Death	Concl	Prolif	Death	Concl	Prolif	Death	Concl	Prolif	Death	Concl
1	104	79	Prolif	20	43	Death	43.5	84.3	Death	23	76	Death	33.3	63.3	Death
2	27	10	Prolif	5	5	Death	55.6	83.3	Death	6	6	Death	50.0	66.7	Death
3	1,091	737	Prolif	171	443	Death	27.0	83.9	Death	198	723	Death	23.0	71.2	Death
4	1,211	1,699	Death	222	1,156	Death	32.8	86.8	Death	302	2,324	Death	28.1	70.5	Death
5	550	264	Prolif	100	132	Death	43.1	79.5	Death	133	232	Death	40.5	61.4	Death
6	827	516	Prolif	171	269	Death	42.9	78.4	Death	229	480	Death	38.6	61.9	Death
7	680	351	Prolif	209	171	Prolif	58.9	73.4	Death	297	268	Prolif	51.5	61.9	Death
8	1,426	1,487	Death	214	747	Death	38.9	79.0	Death	282	1,340	Death	35.6	59.8	Death
9	522	412	Prolif	95	192	Death	35.1	74.4	Death	116	295	Death	27.8	65.8	Death
10	3,085	2,644	Prolif	611	1,205	Death	40.1	72.5	Death	791	2,427	Death	32.8	54.8	Death
11	120	143	Death	22	89	Death	32.4	76.1	Death	29	151	Death	28.2	68.9	Death
12	920	455	Prolif	256	227	Prolif	51.1	72.1	Death	327	353	Death	44.0	59.8	Death
13	534	168	Prolif	161	74	Prolif	59.2	66.1	Death	204	115	Prolif	45.0	49.8	Death
14	179	60	Prolif	35	15	Prolif	44.9	71.4	Death	44	26	Prolif	44.0	68.4	Death
15	2,540	1,571	Prolif	306	349	Death	44.8	53.5	Death	387	497	Death	41.2	49.3	Death
16	187	381	Death	42	220	Death	40.0	83.0	Death	57	505	Death	32.4	61.9	Death
17	1,674	1,039	Prolif	486	677	Death	44.3	79.1	Death	668	1,307	Death	35.7	62.1	Death
18	454	602	Death	46	387	Death	16.2	86.4	Death	51	656	Death	13.1	72.6	Death
19	272	210	Prolif	32	115	Death	21.2	79.9	Death	40	202	Death	20.8	73.7	Death
20	67	47	Prolif	29	27	Prolif	58.0	64.3	Death	48	69	Death	58.5	43.1	Prolif
21	617	611	Prolif	130	379	Death	42.1	80.1	Death	171	771	Death	36.7	62.0	Death
22	684	458	Prolif	188	172	Prolif	60.3	64.9	Death	254	277	Death	51.6	49.2	Prolif
23	9,517	7,814	Prolif	1,694	4,408	Death	35.3	76.7	Death	2,101	7,664	Death	29.8	60.6	Death
24	568	226	Prolif	97	81	Prolif	46.9	68.6	Death	114	100	Prolif	41.5	57.5	Death
25	240	325	Death	33	237	Death	18.2	86.5	Death	38	477	Death	12.6	67.8	Death
26	734	397	Prolif	164	182	Death	45.8	70.5	Death	213	348	Death	37.6	58.4	Death
27	7	9	Death	3	5	Death	100.0	71.4	Prolif	6	5	Prolif	75.0	62.5	Prolif
	23,558	18,284	Prolif	5,287	11,604	Death	38.7	76.7	Death	7,129	21,694	Death	33.4	61.2	Death

Table 5 summarizes the analysis conducted for chemical 17 for all the chemicals. If the number of abstracts that report cell proliferation versus cell death is used as a proxy for association, a decision maker would conclude that 20/27 chemicals are more associated with proliferation than death. However, if the number of abstracts that show an increase in cell proliferation or death (i.e. that had supporting evidence) was used, the decision would change from proliferation to death for 13 of the 27 chemicals. If instead the percentage of abstracts that had supporting evidence was considered, the decision would change for 21 chemicals (20 from proliferation to death and 1 from death to proliferation). If instead the number of claims rather than the number of abstracts was used, the decision would change for 17 chemicals (16 from proliferation to death and 1 from death to proliferation) and if the percentage of claims was used the decision would change 19 times (18 from proliferation to death and 1 from death to proliferation). The overall choice would also change from proliferation to death regardless of which claim measure was used. These results suggest that authors of automated systems should specify which step of the information synthesis process is being automated and potentially a caution to readers that simplyreporting an outcome should not be interpreted as an association (either positive or negative).

Although the proposed approach moves us closer to the manual risk assessment process, there are other tasks in a systematic review process that are not part of this system. For example, decision makers still need to search the grey literature (studies conducted but not published) and follow references to minimize bias (the latter is a candidate for automation). Similarly, no attempt is made to assess the quality of the study which is required in human systematic reviews [2]; however, it would seem that further work in this regard is needed for systematic reviews involving animal studies where 71% of preclinical systematic reviews did not assess the methodological quality [43]. Understanding how a stressor impacts the cell cycle is just one of the many outcomes that a decision maker would consider when establishing public policy around potential carcinogens but cellular level outcomes are just one of the many streams of evidence that includes amongst other endpoints genetic markers, and evidence on humans and animals is weighted treated differently when determining if there is a sufficient amount of evidence to change policy. We also do not attempt to differentiate between major and minor claims, however human inter-rater reliability to establish this distinction has been reported as low [26].

In addition to providing insight about outcomes for risk assessments, this approach may also contribute to discussions around publication bias and the way in which authors choose to describe their findings. The search criterion used in this study considered only the chemical name, but the chemicals considered were selected because of their potential role in cancer. Against that backdrop cell proliferation might be considered a negative outcome (i.e. that cancer is progressing) whereas cell death might be a positive outcome (i.e. that the cancer progression has been halted). This might influence an author’s preference to frame the negative outcome (proliferation) using refuting evidence and the positive outcome (death) using supporting evidence. It is notable that authors use more neutral claims when reporting cells in general that are neither favorable nor unfavorable. Further work is needed to unpack the relationship between framing and the directionality that authors use when reporting outcomes.

## Conclusions

Public policy regarding chemicals takes place against a complex backdrop of legal regulations such as Section 6(b) of the Toxic Substances Control Act (TSCA) in the US, and the Regulation No 1907/2006 concerning the Registration, Evaluation, Authorisation and Restriction of Chemicals (REACH), efforts in the EU. Regardless of the statutory requirements, the human processes used to synthesize evidence can slow down efforts to update public policy and do not scale to cumulative risk assessments where multiple stressors are considered. Automated systems that augment human efforts are urgently needed, but such techniques will only be adopted if they are accurate and consistent with the level of transparency needed in this setting.

The approach introduced in this paper combines domain expertise to clearly articulate target outcomes, knowledge resources to capture target outcomes, and natural language processing methods to overcome surface level differences between how a target outcome is represented in a formal ontology and how those same concepts are reported in the scientific literature. To be consistent with the manual efforts used to conduct a chemical risk assessment, the search strategy, and the inclusion and exclusion criterion must also be explicit. In contrast to work that automates the retrieval step of the information synthesis process, the approach presented here automates the extraction step and provides decision makers with a visualization using waffle plots that reflect the distribution of supporting, neutral, and refuting evidence for a given outcome. This is consistent with a fundamental tenant of a systematic review where all evidence is provided to a reader, not just the evidence that supports an author’s position.

Experiments using 482K abstracts for 27 chemicals show that refuting evidence (where the target outcome has decreased) was higher for cell proliferation (45.9%) and general cell changes (37.7%) than for cell death (25.0%), moreover that only cell death had more supporting claims (61.3%). If the number of abstracts that measure an outcome was used as a proxy for association there would be a stronger association with cell proliferation than cell death (20/27 chemicals). However, if the amount of supporting evidence was used (that the outcome increased) the conclusion would change for 21 of the 27 chemicals—20 from proliferation to death and 1 from death to proliferation. This suggests that results from the retrieval step (i.e. the number of abstracts that measure an outcome) can be misleading if not accompanied with results from the extraction step where the directionality of the outcome is established.

## Supporting information

S1 Appendix Exact search strings used in PubMed search for each of the 27 chemicals. (DOCX)

S2 Appendix Data used in Figs 6–8. (XLSX)
