# Supplementary material for: Using semantics to scale up evidence-based chemical risk-assessments
Source: PLoS One. 2021 Dec 15;16(12):e0260712. doi: 10.1371/journal.pone.0260712 (PMC8673667; doi:10.1371/journal.pone.0260712)
Supplement: S1 Appendix — (DOCX) [file pone.0260712.s001.docx]

# Appendix A - search string

A set of 27 chemicals were included in the second search strategy. For each chemical, an expert reviewed the synonyms from pubchem that were included in the query along with the constraints were applied to the breast cancer collection to include only those abstracts that reported original, were published in English and included abstract text. The chemicals and corresponding search terms are listed below.

## 1,3-BUTADIENE

(1,3-BUTADIENE[MeSH Terms] OR "1,3-BUTADIENE"[All Fields] OR “Buta-1,3-diene"[MeSH Terms] OR "Buta-1,3-diene"[All Fields] OR “BUTADIENE"[MeSH Terms] OR "BUTADIENE"[All Fields] OR “Vinylethylene"[MeSH Terms] OR "Vinylethylene"[All Fields] OR “Biethylene"[MeSH Terms] OR "Biethylene"[All Fields] OR “Erythrene"[MeSH Terms] OR “Erythrene"[All Fields] OR “Bivinyl"[MeSH Terms] OR "Bivinyl"[All Fields] OR “Pyrrolylene"[MeSH Terms] OR "Pyrrolylene"[All Fields] OR “1,3 butadiene"[MeSH Terms] OR "1,3 butadiene"[All Fields])

## 4-aminobiphenyl

(4-Aminobiphenyl[MeSH] OR "4-Aminobiphenyl"[All Fields] OR “4-Phenylaniline"[MeSH Terms] OR "4-Phenylaniline"[All Fields] OR “4-Aminodiphenyl"[MeSH Terms] OR "4-Aminodiphenyl"[All Fields] OR “4-BIPHENYLAMINE"[MeSH Terms] OR "4-BIPHENYLAMINE"[All Fields] OR “biphenyl-4-amine"[MeSH Terms] OR "biphenyl-4-amine"[All Fields] OR “[1,1'-Biphenyl]-4-amineMylosar"[MeSH Terms] OR “[1,1'-Biphenyl]-4-amine"[All Fields] OR “Biphenyl-4-ylamine"[MeSH Terms] OR "Biphenyl-4-ylamine"[All Fields] OR “Xenylamine"[MeSH Terms] OR "Xenylamine"[All Fields] OR “4-Biphenylylamine"[MeSH Terms] OR "4-Biphenylylamine"[All Fields] OR “p-Phenylaniline"[MeSH Terms] OR "p-Phenylaniline"[All Fields] OR “p-Aminobiphenyl"[MeSH Terms] OR "p-Aminobiphenyl"[All Fields] OR “p-Aminodiphenyl"[MeSH Terms] OR "p-Aminodiphenyl"[All Fields] OR “p-Biphenylamine"[MeSH Terms] OR "p-Biphenylamine"[All Fields] OR “p-Xenylamine"[MeSH Terms] OR "p-Xenylamine"[All Fields] OR “Biphenylamine"[MeSH Terms] OR "Biphenylamine"[All Fields] OR “Paraaminodiphenyl"[MeSH Terms] OR "Paraaminodiphenyl"[All Fields] OR “Xenylamin"[MeSH Terms] OR "Xenylamin"[All Fields] OR “4-Aminodifenil"[MeSH Terms] OR "4-Aminodifenil"[All Fields] OR “4-Amino-1,1'-biphenyl"[MeSH Terms] OR "4-Amino-1,1'-biphenyl"[All Fields] OR “4-Amino-1,1'-biphenyl"[MeSH Terms] OR "4-Amino-1,1'-biphenyl"[All Fields] OR “4-Aminobifenyl"[MeSH Terms] OR "4-Aminobifenyl"[All Fields] OR “4-Bifenylamin"[MeSH Terms] OR "4-Bifenylamin"[All Fields] OR “4-phenylphenylamine"[MeSH Terms] OR "4-phenylphenylamine"[All Fields] OR “p-Amino diphenyl"[MeSH Terms] OR "p-Amino diphenyl"[All Fields] OR “para-aminobiphenyl"[MeSH Terms] OR "para-aminobiphenyl"[All Fields] OR “4-amino-biphenyl"[MeSH Terms] OR "4-amino-biphenyl"[All Fields] OR “4-phenyl aniline"[MeSH Terms] OR "4-phenyl aniline"[All Fields] OR “4-Phenylbenzenamine"[MeSH Terms] OR "4-Phenylbenzenamine"[All Fields])

## 5-azacytidine

(5-azacytidine[MeSH Terms] OR "5-azacytidine"[All Fields] OR “Azacitidine"[MeSH Terms] OR "Azacitidine"[All Fields] OR “Ladakamycin"[MeSH Terms] OR "Ladakamycin"[All Fields] OR “Azacytidine"[MeSH Terms] OR "Azacytidine"[All Fields] OR “Vidaza"[MeSH Terms] OR "Vidaza"[All Fields] OR “Mylosar"[MeSH Terms] OR “Mylosar"[All Fields] OR “Azacitidina"[MeSH Terms] OR "Azacitidina"[All Fields] OR “Azacitidinum"[MeSH Terms] OR "Azacitidinum"[All Fields] OR “5-azacitidine"[MeSH Terms] OR "5-azacitidine"[All Fields])

## Arsenic

(Arsenic[MeSH Terms] OR "Arsenic"[All Fields] OR “Arsenic compounds"[MeSH Terms] OR "Arsenic compounds"[All Fields] OR “Arsenic, inorganic"[MeSH Terms] OR "Arsenic, inorganic"[All Fields] OR “Arsenic, elemental"[MeSH Terms] OR " Arsenic, elemental"[All Fields] OR “Arsenic powder"[MeSH Terms] OR "Arsenic powder"[All Fields])

## Asbestos

(asbestos[MeSH] OR "asbestos"[All Fields] OR “asbestos"[MeSH Terms] OR "asbestos"[All Fields])

## benzo[a]pyrene

(benzo[a]pyrene [MeSH Terms] OR "benzo[a]pyrene"[All Fields] OR “3,4-Benzopyrene"[MeSH Terms] OR "3,4-Benzopyrene"[All Fields] OR “BENZO(A)PYRENE"[MeSH Terms] OR "BENZO(A)PYRENE"[All Fields] OR “6,7-Benzopyrene"[MeSH Terms] OR "6,7-Benzopyrene"[All Fields] OR “Benz[a]pyrene"[MeSH Terms] OR "Benz[a]pyrene"[All Fields] OR “3,4 Benzpyrene"[MeSH Terms] OR “3,4 Benzpyrene"[All Fields] OR “3,4-Benz(a)pyrene"[MeSH Terms] OR "3,4-Benz(a)pyrene"[All Fields] OR “3,4-Benzo(a)pyrene"[MeSH Terms] OR "3,4-Benzo(a)pyrene"[All Fields] OR “Benzo[a]pyrene (BaP)"[MeSH Terms] OR "Benzo[a]pyrene (BaP)"[All Fields])

## bisphenol A

(Bisphenol A[MeSH Terms] OR "Bisphenol A"[All Fields] OR “2,2-Bis(4-hydroxyphenyl)propane"[MeSH Terms] OR "2,2-Bis(4-hydroxyphenyl)propane"[All Fields] OR “Bisphenol-A"[MeSH Terms] OR "Bisphenol-A"[All Fields] OR “BPA"[MeSH Terms] OR "BPA"[All Fields] OR “Bisphenol A."[MeSH Terms] OR " Bisphenol A."[All Fields])

## Cadmium

(Cadmium[MeSH Terms] OR "Cadmium"[All Fields] OR “Cadmium, elemental"[MeSH Terms] OR "Cadmium, elemental"[All Fields] OR “Cadmium [Cadmium and cadmium compounds]"[MeSH Terms] OR "Cadmium [Cadmium and cadmium compounds]"[All Fields])

## Chloroform

(CHLOROFORM [MeSH Terms] OR "CHLOROFORM"[All Fields] OR “Trichloromethane"[MeSH Terms] OR "Trichloromethane"[All Fields] OR “Formyl trichloride"[MeSH Terms] OR "Formyl trichloride"[All Fields] OR “Methane, trichloro-"[MeSH Terms] OR "Methane, trichloro-"[All Fields] OR “Trichloroform"[MeSH Terms] OR "Trichloroform"[All Fields] OR “Methane trichloride"[MeSH Terms] OR “Methane trichloride"[All Fields] OR “Methenyl trichloride"[MeSH Terms] OR "Methenyl trichloride"[All Fields] OR “Methyl trichloride"[MeSH Terms] OR "Methyl trichloride"[All Fields] OR “chloroforrn"[MeSH Terms] OR "chloroforrn"[All Fields] OR “trichlormethane"[MeSH Terms] OR "trichlormethane"[All Fields])

## Cyclosporine

(cyclosporin A[MeSH] OR " cyclosporin A"[All Fields] OR “cyclosporine"[MeSH Terms] OR "cyclosporine"[All Fields] OR “Ciclosporin"[MeSH Terms] OR "Ciclosporin"[All Fields] OR “Cyclosporine A"[MeSH Terms] OR "Cyclosporine A"[All Fields] OR “Ciclosporine"[MeSH Terms] OR "Ciclosporine"[All Fields] OR “Cyclosporin"[MeSH Terms] OR “Cyclosporin"[All Fields] OR “Ciclosporina"[MeSH Terms] OR "Ciclosporina"[All Fields] OR “Ciclosporinum"[MeSH Terms] OR " Ciclosporinum"[All Fields] OR “CyclosporinA"[MeSH Terms] OR “CyclosporinA"[All Fields])

## Dichloroacetate

(Dichloroacetate[MeSH] OR " Dichloroacetate"[All Fields] OR “2,2-dichloroacetate"[MeSH Terms] OR "2,2-dichloroacetate"[All Fields] OR “Dichloracetate"[MeSH Terms] OR "Dichloracetate"[All Fields]

OR “2,2-bis(chloranyl)ethanoate"[MeSH Terms] OR "2,2-bis(chloranyl)ethanoate"[All Fields])

## diethylnitrosamine

(N-NITROSODIETHYLAMINE [MeSH Terms] OR "N-NITROSODIETHYLAMINE"[All Fields] OR “Diethylnitrosamine"[MeSH Terms] OR "Diethylnitrosamine"[All Fields] OR “Diethylnitrosoamine"[MeSH Terms] OR "Diethylnitrosoamine"[All Fields] OR “N-Ethyl-N-nitrosoethanamine"[MeSH Terms] OR "N-Ethyl-N-nitrosoethanamine"[All Fields] OR “N,N-Diethylnitrosamine"[MeSH Terms] OR "N,N-Diethylnitrosamine"[All Fields] OR “N,N-Diethylnitrosoamine"[MeSH Terms] OR “N,N-Diethylnitrosoamine"[All Fields] OR “Nitrosodiethylamine"[MeSH Terms] OR "Nitrosodiethylamine"[All Fields] OR “Ethanamine, N-ethyl-N-nitroso-"[MeSH Terms] OR "Ethanamine, N-ethyl-N-nitroso-"[All Fields] OR “Diethylnitrosamide"[MeSH Terms] OR "Diethylnitrosamide"[All Fields] OR “Diethylamine, N-nitroso-"[MeSH Terms] OR "Diethylamine, N-nitroso-"[All Fields] OR “N-Nitroso-N,N-diethylamine"[MeSH Terms] OR "N-Nitroso-N,N-diethylamine"[All Fields] OR “N,N-diethylnitrous amide"[MeSH Terms] OR "N,N-diethylnitrous amide"[All Fields] OR “RCRA waste number U174"[MeSH Terms] OR "RCRA waste number U174"[All Fields] OR “N-Diethylnitrosamine"[MeSH Terms] OR "N-Diethylnitrosamine"[All Fields] OR “Nitrosamine, diethyl-"[MeSH Terms] OR "Nitrosamine, diethyl-"[All Fields] OR “N-Nitroso-diaethylamine"[MeSH Terms] OR "N-Nitroso-diaethylamine"[All Fields] OR “N-Ethyl-N-nitroso-ethanamine"[MeSH Terms] OR "N-Ethyl-N-nitroso-ethanamine"[All Fields] OR “1,1-Diethyl-2-oxohydrazine"[MeSH Terms] OR "1,1-Diethyl-2-oxohydrazine"[All Fields] OR “N-Nitrosodiethlamine"[MeSH Terms] OR "N-Nitrosodiethlamine"[All Fields] OR “n-nitroso-diethylamine"[MeSH Terms] OR "n-nitroso-diethylamine"[All Fields])

## diethylstilbestrol

(diethylstilbestrol [MeSH Terms] OR "diethylstilbestrol"[All Fields] OR “Stilbestrol"[MeSH Terms] OR "Stilbestrol"[All Fields] OR “Stilboestrol"[MeSH Terms] OR "Stilboestrol"[All Fields] OR “Distilbene"[MeSH Terms] OR "Distilbene"[All Fields] OR “Stilbetin"[MeSH Terms] OR "Stilbetin"[All Fields] OR “Diethylstilbesterol"[MeSH Terms] OR “Diethylstilbesterol"[All Fields] OR “Dietilestilbestrol"[MeSH Terms] OR "Dietilestilbestrol"[All Fields] OR “Diethylstilboesterol"[MeSH Terms] OR "Diethylstilboesterol"[All Fields] OR “Diethyl stilbestrol"[MeSH Terms] OR "Diethyl stilbestrol"[All Fields])

## Ethylene oxide

(Oxirane[MeSH] OR "Oxirane"[All Fields] OR “ETHYLENE OXIDE"[MeSH Terms] OR "ETHYLENE OXIDE"[All Fields] OR “1,2-Epoxyethane"[MeSH Terms] OR "1,2-Epoxyethane"[All Fields] OR “Oxacyclopropane"[MeSH Terms] OR "Oxacyclopropane"[All Fields] OR “Ethene oxide"[MeSH Terms] OR "Ethene oxide"[All Fields] OR “Dimethylene oxide"[MeSH Terms] OR “Dimethylene oxide"[All Fields] OR “Amprolene"[MeSH Terms] OR "Amprolene"[All Fields] OR “Anprolene"[MeSH Terms] OR "Anprolene"[All Fields] OR “Anproline"[MeSH Terms] OR "Anproline"[All Fields] OR “Dihydrooxirene"[MeSH Terms] OR "Dihydrooxirene"[All Fields] OR “Oxidoethane"[MeSH Terms] OR "Oxidoethane"[All Fields] OR “Oxyfume"[MeSH Terms] OR "Oxyfume"[All Fields] OR “Aethylenoxid"[MeSH Terms] OR "Aethylenoxid"[All Fields])

## Formaldehyde

(formaldehyde[MeSH] OR "formaldehyde"[All Fields] OR “formalin"[MeSH Terms] OR "formalin"[All Fields] OR “Polyformaldehyde"[MeSH Terms] OR "Polyformaldehyde"[All Fields])

## fumonisin b1

(fumonisin b1[MeSH Terms] OR "fumonisin b1"[All Fields] OR “Macrofusine"[MeSH Terms] OR "Macrofusine"[All Fields] OR “fumonisin-B1"[MeSH Terms] OR "fumonisin-B1"[All Fields] OR “fumonisin B(1)"[MeSH Terms] OR "fumonisin B(1)"[All Fields])

## Genistein

(genistein[MeSH Terms] OR "genistein"[All Fields] OR “Prunetol"[MeSH Terms] OR "Prunetol"[All Fields] OR “4',5,7-Trihydroxyisoflavone"[MeSH Terms] OR "4',5,7-Trihydroxyisoflavone"[All Fields] OR “Genisteol"[MeSH Terms] OR "Genisteol"[All Fields] OR “Genisterin"[MeSH Terms] OR "Genisterin"[All Fields] OR “Sophoricol"[MeSH Terms] OR "Sophoricol"[All Fields] OR “5,7,4'-Trihydroxyisoflavone"[MeSH Terms] OR "5,7,4'-Trihydroxyisoflavone"[All Fields] OR “5,7-dihydroxy-3-(4-hydroxyphenyl)-4H-chromen-4-one"[MeSH Terms] OR "5,7-dihydroxy-3-(4-hydroxyphenyl)-4H-chromen-4-one"[All Fields] OR “Bonistein"[MeSH Terms] OR "Bonistein"[All Fields] OR “Genestein"[MeSH Terms] OR "Genestein"[All Fields] OR “Differenol A"[MeSH Terms] OR "Differenol A"[All Fields])

## Irinotecan

(irinotecan[MeSH] OR "irinotecan"[All Fields] OR “Irinotecanum"[MeSH Terms] OR "Irinotecanum"[All Fields])

## Methylene chloride

(DICHLOROMETHANE[MeSH] OR "DICHLOROMETHANE"[All Fields] OR “Methylene chloride"[MeSH Terms] OR "Methylene chloride"[All Fields] OR “Methylene dichloride"[MeSH Terms] OR "Methylene dichloride"[All Fields] OR “Methane, dichloro-"[MeSH Terms] OR "Methane, dichloro-"[All Fields] OR “Methylene bichloride"[MeSH Terms] OR "Methylene bichloride"[All Fields] OR “Methane dichloride"[MeSH Terms] OR “Methane dichloride"[All Fields] OR “methlyenechloride"[MeSH Terms] OR "methlyenechloride"[All Fields] OR “methylenechloride"[MeSH Terms] OR "methylenechloride"[All Fields] OR “methlene chloride"[MeSH Terms] OR "methlene chloride"[All Fields] OR “methyene chloride"[MeSH Terms] OR "methyene chloride"[All Fields] OR “methylen chloride"[MeSH Terms] OR "methylen chloride"[All Fields] OR “methlyene chloride"[MeSH Terms] OR "methlyene chloride"[All Fields] OR “methylene,chloride"[MeSH Terms] OR "methylene,chloride"[All Fields])

## Nafenopin

(Nafenopin[MeSH Terms] OR "Nafenopin"[All Fields] OR “Nafenoic acid"[MeSH Terms] OR "Nafenoic acid"[All Fields] OR “2-Methyl-2-(4-(1,2,3,4-tetrahydronaphthalen-1-yl)phenoxy)propanoic acid"[MeSH Terms] OR "2-Methyl-2-(4-(1,2,3,4-tetrahydronaphthalen-1-yl)phenoxy)propanoic acid"[All Fields] OR “2-Methyl-2-(4-(1,2,3,4-tetrahydro-1-naphthyl)phenoxy)propanoic acid"[MeSH Terms] OR "2-Methyl-2-(4-(1,2,3,4-tetrahydro-1-naphthyl)phenoxy)propanoic acid"[All Fields] OR “2-Methyl-2-(p-(1,2,3,4-tetrahydro-1-naphthyl)phenoxy)propionic acid"[MeSH Terms] OR "2-Methyl-2-(p-(1,2,3,4-tetrahydro-1-naphthyl)phenoxy)propionic acid"[All Fields] OR “2-Methyl-2-(4-(1,2,3,4-tetrahydro-1-naphthalenyl)phenoxy)propanoic acid"[MeSH Terms] OR “2-Methyl-2-(4-(1,2,3,4-tetrahydro-1-naphthalenyl)phenoxy)propanoic acid"[All Fields] OR “2-methyl-2-[4-(1,2,3,4-tetrahydronaphthalen-1-yl)phenoxy]propanoic acid"[MeSH Terms] OR "2-methyl-2-[4-(1,2,3,4-tetrahydronaphthalen-1-yl)phenoxy]propanoic acid"[All Fields] OR “2-methyl-2-{[4-(1,2,3,4-tetrahydronaphthalen-1-yl)phenyl]oxy}propanoic acid"[MeSH Terms] OR "2-methyl-2-{[4-(1,2,3,4-tetrahydronaphthalen-1-yl)phenyl]oxy}propanoic acid"[All Fields])

## Okadaic acid

(okadaic acid[MeSH] OR "okadaic acid"[All Fields] OR “Ocadaic Acid"[MeSH Terms] OR "Ocadaic Acid"[All Fields])

## phenobarnital

(phenobarbital[MeSH Terms] OR "phenobarbital"[All Fields] OR “Phenobarbitone"[MeSH Terms] OR "Phenobarbitone"[All Fields] OR “Phenobarbitol"[MeSH Terms] OR "Phenobarbitol"[All Fields] OR “Phenylethylbarbiturate"[MeSH Terms] OR "Phenylethylbarbituratel"[All Fields] OR “Phenobarbituric acid"[MeSH Terms] OR "Phenobarbituric acid"[All Fields] OR “Fenobarbital"[MeSH Terms] OR "Fenobarbital"[All Fields] OR “Phenemal"[MeSH Terms] OR "Phenemal"[All Fields] OR “Adonal"[MeSH Terms] OR "Adonal"[All Fields] OR “Phenylethylbarbituric acid"[MeSH Terms] OR "Phenylethylbarbituric acid"[All Fields] OR “Nunol"[MeSH Terms] OR "Nunol"[All Fields] OR “Phenylethylmalonylurea"[MeSH Terms] OR "Phenylethylmalonylurea"[All Fields] OR “Neurobarb"[MeSH Terms] OR "Neurobarb"[All Fields] OR “Phenaemal"[MeSH Terms] OR "Phenaemal"[All Fields] OR “Dormiral"[MeSH Terms] OR "Dormiral"[All Fields] OR “Gardenal"[MeSH Terms] OR "Gardenal"[All Fields] OR “Hysteps"[MeSH Terms] OR "Hysteps"[All Fields] OR “Aphenylbarbi"[MeSH Terms] OR "Aphenylbarbi"[All Fields] OR “Aphenyletten"[MeSH Terms] OR "Aphenyletten"[All Fields] OR “Dezibarbitur"[MeSH Terms] OR "Dezibarbitur"[All Fields] OR “Lepinaletten"[MeSH Terms] OR "Lepinaletten"[All Fields] OR “Lumofridetten”[MeSH Terms] OR "Lumofridettenl"[All Fields] OR “Aephenal"[MeSH Terms] OR "Aephenal"[All Fields] OR “Agrypnal"[MeSH Terms] OR "Agrypnal"[All Fields] OR “Amylofenel"[MeSH Terms] OR "Amylofene"[All Fields] OR “Barbenyl"[MeSH Terms] OR "Barbenyl"[All Fields] OR “Barbiphenyl"[MeSH Terms] OR "Barbiphenyl"[All Fields] OR “Barbipil"[MeSH Terms] OR "Barbipil"[All Fields] OR “Barbivis"[MeSH Terms] OR “Barbivis"[All Fields] OR “Barbonal"[MeSH Terms] OR "Barbonal"[All Fields] OR “Barbophen"[MeSH Terms] OR "Barbophen"[All Fields] OR “Bialminal"[MeSH Terms] OR "Bialminal"[All Fields] OR “Cabronal"[MeSH Terms] OR "Cabronal"[All Fields] OR “Calmetten"[MeSH Terms] OR "Calmetten"[All Fields] OR “Calminal"[MeSH Terms] OR "Calminal"[All Fields] OR “Cardenal"[MeSH Terms] OR "Cardenal"[All Fields] OR “Codibarbita”[MeSH Terms] OR "Codibarbita"[All Fields] OR “Coronaletta"[MeSH Terms] OR "Coronaletta"[All Fields] OR “Cratecil"[MeSH Terms] OR "Cratecil"[All Fields] OR “Doscalun"[MeSH Terms] OR "Doscalun"[All Fields] OR “Ensobarb"[MeSH Terms] OR "Ensobarb"[All Fields] OR “Ensodorm”[MeSH Terms] OR "Ensodorm"[All Fields] OR “Episedal "[MeSH Terms] OR "Episedal "[All Fields] OR “Epsylone"[MeSH Terms] OR "Epsylone"[All Fields] OR “Eskabarb"[MeSH Terms] OR "Eskabarb"[All Fields] OR “Fenbital"[MeSH Terms] OR “Fenbital"[All Fields] OR “Fenylettae"[MeSH Terms] OR "Fenylettae"[All Fields] OR “Gardepanyl"[MeSH Terms] OR "Gardepanyl"[All Fields] OR “Glysoletten "[MeSH Terms] OR "Glysoletten "[All Fields] OR “Haplopan"[MeSH Terms] OR "Haplopan"[All Fields] OR “Hennoletten"[MeSH Terms] OR "Hennoletten"[All Fields] OR “Hypnaletten"[MeSH Terms] OR "Hypnaletten"[All Fields] OR “Hypnette"[MeSH Terms] OR "Hypnette"[All Fields] OR “Hypnogen"[MeSH Terms] OR "Hypnogen"[All Fields] OR “Hypnolone"[MeSH Terms] OR "Hypnolone"[All Fields] OR “Hypnoltol"[MeSH Terms] OR "Hypnoltol"[All Fields] OR “Liquital"[MeSH Terms] OR "LiquitalAzacitidinum"[All Fields] OR “Lixophen"[MeSH Terms] OR "Lixophen"[All Fields] OR “Lubergal"[MeSH Terms] OR "Lubergal"[All Fields] OR “Lubrokal"[MeSH Terms] OR "Lubrokal"[All Fields] OR “Lumesettes"[MeSH Terms] OR "Lumesettes"[All Fields] OR “Luphenil”[MeSH Terms] OR "Luphenil"[All Fields] OR “Nirvonal"[MeSH Terms] OR “Nirvonal"[All Fields] OR “Parkotal"[MeSH Terms] OR "Parkotal"[All Fields] OR “Pharmetten"[MeSH Terms] OR "Pharmetten"[All Fields] OR “Phenemalum"[MeSH Terms] OR "Phenemalum"[All Fields] OR “Phenobal "[MeSH Terms] OR "Phenobal"[All Fields] OR “Phenobarbyl"[MeSH Terms] OR "Phenobarbyl"[All Fields] OR “Phenoluric"[MeSH Terms] OR "Phenoluric"[All Fields] OR “Phenolurio"[MeSH Terms] OR "Phenolurio"[All Fields] OR “Phenomet"[MeSH Terms] OR "Phenomet"[All Fields] OR “Phenonyl"[MeSH Terms] OR "Phenonyl"[All Fields] OR “Phenoturic"[MeSH Terms] OR "Phenoturic"[All Fields] OR “Phenyletten"[MeSH Terms] OR "Phenyletten"[All Fields] OR “Phenyral"[MeSH Terms] OR "Phenyral"[All Fields] OR “Polcominal"[MeSH Terms] OR "Polcominal"[All Fields] OR “Promptonal"[MeSH Terms] OR "Promptonal"[All Fields] OR “Sedizorin"[MeSH Terms] OR "Sedizorin"[All Fields] OR “Sedonettes"[MeSH Terms] OR "Sedonettes"[All Fields] OR “Solfoton"[MeSH Terms] OR "Solfoton"[All Fields] OR “Sombutol"[MeSH Terms] OR "Sombutol"[All Fields] OR “Somnolens”[MeSH Terms] OR "Somnolens"[All Fields] OR “Somnoletten"[MeSH Terms] OR "Somnoletten"[All Fields] OR “Somnosan"[MeSH Terms] OR "Somnosan"[All Fields] OR “Spasepilin"[MeSH Terms] OR "Spasepilin"[All Fields] OR “Starifen"[MeSH Terms] OR "Starifen"[All Fields] OR “Starilettae"[MeSH Terms] OR "Starilettae"[All Fields] OR “Teolaxin"[MeSH Terms] OR "Teolaxin"[All Fields] OR “Barbita"[MeSH Terms] OR "Barbita"[All Fields] OR “Bardorm"[MeSH Terms] OR "Bardorm"[All Fields] OR “Bartol"[MeSH Terms] OR "Bartol"[All Fields] OR “Chinoin"[MeSH Terms] OR "Chinoin"[All Fields] OR “Duneryl"[MeSH Terms] OR "Duneryl"[All Fields] OR “Epanal"[MeSH Terms] OR “Epanal"[All Fields] OR “Epidorm"[MeSH Terms] OR "Epidorm"[All Fields] OR “Epilol"[MeSH Terms] OR "Epilol"[All Fields] OR “Etilfen"[MeSH Terms] OR "Etilfen"[All Fields] OR “Euneryl”[MeSH Terms] OR "Euneryl"[All Fields] OR “Fenemal"[MeSH Terms] OR "Fenemal"[All Fields] OR “Fenosed"[MeSH Terms] OR "Fenosed"[All Fields] OR “Haplos"[MeSH Terms] OR "Haplos"[All Fields] OR “Henotal”[MeSH Terms] OR "Henotal"[All Fields] OR “Leonal"[MeSH Terms] OR "Leonal"[All Fields] OR “Lepinal"[MeSH Terms] OR "Lepinal"[All Fields] OR “Linasen"[MeSH Terms] OR "Linasen"[All Fields] OR “Lumesyn"[MeSH Terms] OR "Lumesyn"[All Fields] OR “Luramin"[MeSH Terms] OR "Luramin"[All Fields] OR “Molinal"[MeSH Terms] OR "Molinal"[All Fields] OR “Noptil"[MeSH Terms] OR "Noptil"[All Fields] OR “Sedabar"[MeSH Terms] OR "Sedabar"[All Fields] OR “Sedicat"[MeSH Terms] OR "Sedicat"[All Fields] OR “Sedlyn"[MeSH Terms] OR "Sedlyn"[All Fields] OR “Sedofen"[MeSH Terms] OR "Sedofen"[All Fields] OR “Sedonal"[MeSH Terms] OR "Sedonal"[All Fields] OR “Sevenal"[MeSH Terms] OR "Sevenal"[All Fields] OR “Somonal"[MeSH Terms] OR "Somonal"[All Fields] OR “Seda-Tablinen"[MeSH Terms] OR "Seda-Tablinen"[All Fields] OR “Blu-phen"[MeSH Terms] OR "Blu-phen"[All Fields] OR “Nova-Pheno"[MeSH Terms] OR "Nova-Pheno"[All Fields] OR “Solu-Barb"[MeSH Terms] OR "Solu-Barb"[All Fields] OR “Hypno-Tablinetten"[MeSH Terms] OR "Hypno-Tablinetten"[All Fields] OR “Stental Extentabs”[MeSH Terms] OR "Stental Extentabs"[All Fields] OR “Phen-Bar"[MeSH Terms] OR "Phen-Bar"[All Fields] OR “Phenobarb"[MeSH Terms] OR "Phenobarb"[All Fields] OR “Sedophen"[MeSH Terms] OR "Sedophen"[All Fields] OR “Talpheno"[MeSH Terms] OR "Talpheno"[All Fields] OR “Triabarb"[MeSH Terms] OR "Triabarb"[All Fields] OR “Versomnal"[MeSH Terms] OR "Versomnal"[All Fields] OR “5-Ethyl-5-phenylbarbituric acid"[MeSH Terms] OR "5-Ethyl-5-phenylbarbituric acid"[All Fields] OR “5-Phenyl-5-ethylbarbituric acid"[MeSH Terms] OR "5-Phenyl-5-ethylbarbituric acid"[All Fields] OR “Tridezibarbitur"[MeSH Terms] OR "Tridezibarbitur"[All Fields] OR “Triphenatol"[MeSH Terms] OR "Triphenatol"[All Fields] OR “Zadoletten"[MeSH Terms] OR "Zadoletten"[All Fields] OR “Barbinal"[MeSH Terms] OR "Barbinal"[All Fields] OR “Barbiphen"[MeSH Terms] OR "Barbiphen"[All Fields] OR “Damoral"[MeSH Terms] OR "Damoral"[All Fields] OR “Dormina"[MeSH Terms] OR "Dormina"[All Fields] OR “Lefebar"[MeSH Terms] OR "Lefebar"[All Fields] OR “Lephebar"[MeSH Terms] OR "Lephebar"[All Fields] OR “Stental"[MeSH Terms] OR "Stental"[All Fields] OR “Teoloxin"[MeSH Terms] OR " eoloxin"[All Fields] OR “Theoloxin”[MeSH Terms] OR "Theoloxin"[All Fields] OR “Zadonal"[MeSH Terms] OR "Zadonal"[All Fields] OR “SK-Phenobarbital"[MeSH Terms] OR "SK-Phenobarbital"[All Fields] OR “Phenobarbitalum"[MeSH Terms] OR "Phenobarbitalum"[All Fields] OR “Phenobarbitonum"[MeSH Terms] OR "Phenobarbitonum"[All Fields] OR “Thenobarbital"[MeSH Terms] OR "Thenobarbital"[All Fields] OR “Austrominal"[MeSH Terms] OR "Austrominal"[All Fields] OR “5-ethyl-5-phenylpyrimidine-2,4,6(1H,3H,5H)-trione"[MeSH Terms] OR "5-ethyl-5-phenylpyrimidine-2,4,6(1H,3H,5H)-trione"[All Fields] OR “Dormital"[MeSH Terms] OR "Dormital"[All Fields] OR “Fenobarbitale [DCIT]"[MeSH Terms] OR "Fenobarbitale [DCIT]"[All Fields] OR “Elixir of phenobarbital"[MeSH Terms] OR "Elixir of phenobarbital"[All Fields] OR “Phenobarbitalum [INN]"[MeSH Terms] OR "Phenobarbitalum [INN]"[All Fields] OR “Phenylethylbarbitursaeure"[MeSH Terms] OR "Phenylethylbarbitursaeure"[All Fields] OR “Chardonna-2"[MeSH Terms] OR "Chardonna-2"[All Fields] OR “Phenylaethylbarbitursaeure"[MeSH Terms] OR " Phenylaethylbarbitursaeure"[All Fields] OR “2,4,6(1H,3H,5H)-Pyrimidinetrione, 5-ethyl-5-phenyl-"[MeSH Terms] OR "2,4,6(1H,3H,5H)-Pyrimidinetrione, 5-ethyl-5-phenyl-"[All Fields] OR “Phenylethyl barbituric acid"[MeSH Terms] OR "Phenylethyl barbituric acid"[All Fields] OR “Fenobarbital [INN-Spanish]"[MeSH Terms] OR "Fenobarbital [INN-Spanish]"[All Fields]  OR “Phenyl-ethyl-barbituric acid "[MeSH Terms] OR "Phenyl-ethyl-barbituric acid"[All Fields] OR “Phenobarbitalum [INN-Latin]"[MeSH Terms] OR "Phenobarbitalum [INN-Latin]"[All Fields] OR (Barbituric acid, 5-ethyl-5-phenyl-[MeSH] OR "Barbituric acid, 5-ethyl-5-phenyl-"[All Fields] OR “5-Ethyl-5-phenyl-2,4,6(1H,3H,5H)-pyrimidinetrione"[MeSH Terms] OR "5-Ethyl-5-phenyl-2,4,6(1H,3H,5H)-pyrimidinetrione"[All Fields] OR “Barbilehae (barbilettae)"[MeSH Terms] OR "Barbilehae (barbilettae)"[All Fields] OR “5-ethyl-5-phenyl-1,3-diazinane-2,4,6-trione"[MeSH Terms] OR "5-ethyl-5-phenyl-1,3-diazinane-2,4,6-trione"[All Fields] OR “barbapil"[MeSH Terms] OR "barbapil"[All Fields] OR “barbellen"[MeSH Terms] OR "barbellen"[All Fields] OR “barbellon"[MeSH Terms] OR "barbellon"[All Fields] OR “barbilehae"[MeSH Terms] OR "barbilehae"[All Fields] OR “phenylral"[MeSH Terms] OR "phenylral"[All Fields] OR “theominal"[MeSH Terms] OR "theominal"[All Fields] OR “Barbipenyl”[MeSH Terms] OR "Barbipenyl"[All Fields] OR “Barbinol"[MeSH Terms] OR "Barbinol"[All Fields] OR “Donphen"[MeSH Terms] OR "Donphen"[All Fields])

## pyridine

(pyridine[MeSH] OR "pyridine"[All Fields] OR “Pyridin"[MeSH Terms] OR "Pyridin"[All Fields]  OR “Piridina"[MeSH Terms] OR "Piridina"[All Fields]  OR “Pirydyna"[MeSH Terms] OR "Pirydyna"[All Fields] OR “Caswell No. 717"[MeSH Terms] OR "Caswell No. 717"[All Fields] OR “CCRIS 2926"[MeSH Terms] OR "CCRIS 2926"[All Fields] OR “HSDB 118"[MeSH Terms] OR "HSDB 118"[All Fields] OR “CHEBI:16227"[MeSH Terms] OR "CHEBI:16227"[All Fields] OR “FEMA Number 2966"[MeSH Terms] OR "FEMA Number 2966"[All Fields] OR “FEMA No. 2966"[MeSH Terms] OR "FEMA No. 2966"[All Fields] OR “Pentadeuteropyridine"[MeSH Terms] OR "Pentadeuteropyridine"[All Fields])

## styrene

(STYRENE [MeSH Terms] OR "STYRENE"[All Fields] OR “Ethenylbenzene"[MeSH Terms] OR "Ethenylbenzene"[All Fields] OR “Phenylethylene"[MeSH Terms] OR "Phenylethylene"[All Fields] OR “Vinylbenzene"[MeSH Terms] OR "Vinylbenzene"[All Fields] OR “Phenylethene"[MeSH Terms] OR "Phenylethene"[All Fields] OR “Phenethylene"[MeSH Terms] OR “Phenethylene"[All Fields] OR “Styrolene"[MeSH Terms] OR "Styrolene"[All Fields] OR “Vinylbenzol"[MeSH Terms] OR "Vinylbenzol"[All Fields] OR “Vinyl benzene"[MeSH Terms] OR "Vinyl benzene"[All Fields] OR “Styrole"[MeSH Terms] OR "Styrole"[All Fields] OR “Benzene, vinyl-"[MeSH Terms] OR "Benzene, vinyl-"[All Fields] OR “Ethylene, phenyl-"[MeSH Terms] OR "Ethylene, phenyl-"[All Fields] OR “Vinyl-benzene"[MeSH Terms] OR "Vinyl-benzene"[All Fields] OR “phenyl-ethylene"[MeSH Terms] OR "phenyl-ethylene"[All Fields] OR “p-vinyl benzene"[MeSH Terms] OR "p-vinyl benzene"[All Fields])

## Sulindac

(sulindac[MeSH] OR "sulindac"[All Fields] OR “Clinoril"[MeSH Terms] OR "Clinoril"[All Fields] OR “Arthrocine"[MeSH Terms] OR "Arthrocine"[All Fields] OR “Sulindac sulfoxide"[MeSH Terms] OR "Sulindac sulfoxide"[All Fields] OR “Algocetil"[MeSH Terms] OR "Algocetil"[All Fields] OR “Artribid"[MeSH Terms] OR “Artribid"[All Fields] OR “Citireuma"[MeSH Terms] OR "Citireuma"[All Fields] OR “Clisundac"[MeSH Terms] OR "Clisundac"[All Fields] OR “Imbaral"[MeSH Terms] OR "Imbaral"[All Fields] OR “Reumofil"[MeSH Terms] OR "Reumofil"[All Fields] OR “Sulinol"[MeSH Terms] OR "Sulinol"[All Fields] OR “Sudac"[MeSH Terms] OR "Sudac"[All Fields] OR “Arthrobid"[MeSH Terms] OR "Arthrobid"[All Fields] OR “Klinoril"[MeSH Terms] OR "Klinoril"[All Fields] OR “Sulindac (Clinoril)"[MeSH Terms] OR "Sulindac (Clinoril)"[All Fields])

## TCDD

(TCDD[MeSH] OR "TCDD"[All Fields] OR “2,3,7,8-TETRACHLORODIBENZO-P-DIOXIN"[MeSH Terms] OR "2,3,7,8-TETRACHLORODIBENZO-P-DIOXIN"[All Fields] OR “Dioxin"[MeSH Terms] OR "Dioxin"[All Fields] OR “Tetrachlorodibenzodioxin"[MeSH Terms] OR "Tetrachlorodibenzodioxin"[All Fields] OR “2,3,7,8-Tetrachlorodibenzodioxin"[MeSH Terms] OR "2,3,7,8-Tetrachlorodibenzodioxin"[All Fields] OR “2,3,7,8-TCDD"[MeSH Terms] OR "2,3,7,8-TCDD"[All Fields] OR “Tetrachlorodibenzo-p-dioxin"[MeSH Terms] OR "Tetrachlorodibenzo-p-dioxin"[All Fields] OR “2,3,7,8-Tetrachlorodibenzo-1,4-dioxin"[MeSH Terms] OR "2,3,7,8-Tetrachlorodibenzo-1,4-dioxin"[All Fields] OR “Dibenzo-p-dioxin, 2,3,7,8-tetrachloro-"[MeSH Terms] OR "Dibenzo-p-dioxin, 2,3,7,8-tetrachloro-"[All Fields] OR “2,3,7,8-tetrachloro-dibenzo-p-dioxin"[MeSH Terms] OR “2,3,7,8-tetrachloro-dibenzo-p-dioxin"[All Fields] OR “2,3,7,8-Tetrachloro-p-dioxin"[MeSH Terms] OR "2,3,7,8-Tetrachloro-p-dioxin"[All Fields] OR “2,3,7,8-Tetra polychlorinated dibenzo-p-dioxin"[MeSH Terms] OR "2,3,7,8-Tetra polychlorinated dibenzo-p-dioxin"[All Fields])

## Thiobenzamide

(THIOBENZAMIDE[MeSH] OR "THIOBENZAMIDE"[All Fields] OR “Benzothioamide"[MeSH Terms] OR "Benzothioamide"[All Fields] OR “Benzenecarbothioamide"[MeSH Terms] OR "Benzenecarbothioamide"[All Fields] OR “Benzamide, thio-"[MeSH Terms] OR "Benzamide, thio-"[All Fields] OR “Benzothiamide"[MeSH Terms] OR "Benzothiamide"[All Fields] OR “Tiobenzamide"[MeSH Terms] OR "Tiobenzamide"[All Fields] OR “Phenylthioamide"[MeSH Terms] OR "Phenylthioamide"[All Fields])
